# A comparison of prediction approaches for identifying prodromal Parkinson disease

**DOI:** 10.1371/journal.pone.0256592

**Published:** 2021-08-26

**Authors:** Mark N. Warden, Susan Searles Nielsen, Alejandra Camacho-Soto, Roman Garnett, Brad A. Racette

**Affiliations:** 1 Department of Neurology, Washington University School of Medicine, Saint Louis, Missouri, United States of America; 2 Department of Computer Science and Engineering, Washington University in Saint Louis, Saint Louis, Missouri, United States of America; 3 Faculty of Health Sciences, School of Public Heath, University of the Witwatersrand, Johannesburg, South Africa; Vellore Institute of Technology: VIT University, INDIA

## Abstract

Identifying people with Parkinson disease during the prodromal period, including via algorithms in administrative claims data, is an important research and clinical priority. We sought to improve upon an existing penalized logistic regression model, based on diagnosis and procedure codes, by adding prescription medication data or using machine learning. Using Medicare Part D beneficiaries age 66–90 from a population-based case-control study of incident Parkinson disease, we fit a penalized logistic regression both with and without Part D data. We also built a predictive algorithm using a random forest classifier for comparison. In a combined approach, we introduced the probability of Parkinson disease from the random forest, as a predictor in the penalized regression model. We calculated the receiver operator characteristic area under the curve (AUC) for each model. All models performed well, with AUCs ranging from 0.824 (simplest model) to 0.835 (combined approach). We conclude that medication data and random forests improve Parkinson disease prediction, but are not essential.

## Introduction

Parkinson disease (PD) is a progressive, neurodegenerative disorder that is diagnosed when patients experience motor symptoms such as resting tremor, bradykinesia, rigidity, and postural instability. However, before these motor symptoms fully manifest, patients may experience a variety of non-motor symptoms, including cognitive and mood dysfunction, sleep disorders, and varying degrees of autonomic dysfunction [1–5]. This period of disease is termed the “prodromal period” and may provide a critical window of opportunity during which providers could identify PD patients. In particular, earlier recognition of PD might both facilitate the identification of disease-modifying medications, as well as their initiation, when available. Moreover, even without such treatments yet available, earlier identification of PD is essential. During the prodromal disease window, many PD patients experience potentially preventable fall-related morbidity, including substantial excesses of both traumatic brain injuries [6, 7] and fractures [8, 9] relative to comparable individuals without PD.

Towards these ends, researchers have begun to move beyond traditional predictive modeling approaches by applying machine learning methods to a wide variety of data. Several investigators have used machine learning methods to distinguish PD patients from controls, using data obtained from both wearable and non-wearable sensors [10, 11]. While these methods have primarily been used to distinguish newly diagnosed PD patients from controls, other studies were able to distinguish people with potential prodromal PD symptoms, such as hyposmia, from controls [11, 12]. Although these people do have a greater risk of developing PD, this group remains heterogeneous, and there is no “ideal” prodromal PD population. In contrast, retrospective cohort studies using predictor data from the prodromal PD time window afford an opportunity to confirm the PD diagnosis, while providing potentially extensive variables to include in predictive models.

Medicare claims are a rich source of population-based data to predict which patients will be diagnosed eventually with PD. We previously developed a PD predictive model using Medicare claims data, specifically diagnosis and procedure codes, from the five years prior to PD diagnosis [13]. This model contained 536 diagnoses and medical procedures as predictors and achieved an AUC of 0.857, much higher than the AUC of 0.670 achieved with known demographic and medical predictors of PD. At the optimal cut point, sensitivity was 73.5% and specificity was 83.2%. While this least absolute shrinkage and selection operator (LASSO) penalized regression model performed well, the addition of Medicare Part D prescription medication data or the use of other analytic methods, such as machine learning methods, may have the potential to improve model performance. The current study builds upon our previous work by considering whether the addition of prescription medication data improves discrimination and whether a random forest classifier could perform better or help improve the original penalized regression approach [13]. Attempting to improve the model is the logical next step, since we recently validated our original predictive model in a population-based sample followed forward for PD [14]. We hypothesized that inclusion of prescription medication data would improve model performance for four reasons: 1) these medication data offer an alternative way to capture information available from diagnosis codes, which could be incomplete; 2) medication data might provide diagnostic confirmation and evidence of disease severity; 3) medications might serve as proxies for biologic pathways that might be predictive of PD; and 4) some medications might increase or decrease risk of PD, regardless of the indication for the medication, and thus could be independently predictive. Random forest classifiers use a completely different methodology than penalized regression. Therefore, we sought to determine if this innovative approach could outperform or possibly enhance the previous penalized regression model by introducing the probability from the random forest as a predictor in the penalized logistic regression model. We were able to demonstrate modest improvements in model performance.

## Methods

### Standard protocol approvals

This study was approved by the Washington University School of Medicine Human Research Protection Office and the Centers for Medicare and Medicaid Services.

### Study participants

This was a population-based case-control study using Medicare administrative claims data. Briefly, all participants were U.S. residents age 66–90 years old and relying solely on Medicare in 2009. Medicare is the only nationwide health insurance coverage universally available in the U.S., specifically among those age 65 and older. In this age group >98% of Americans participate in Medicare Part A/B, which provides medical coverage. From all of these beneficiaries, we identified those who met all study eligibility criteria (age 66–90, no non-Medicare insurance coverage, and U.S. residence) for the year 2009 using the Medicare “base file.” We then included all incident PD cases and a random sample of comparable beneficiaries as controls who also had Medicare Part D (pharmacy) coverage. We determined PD case status from complete Part A and B Medicare claims data for 2004–2009, with cases identified as having at least one International Classification of Diseases, Ninth Revision, Clinical Modification (ICD9) code for PD (332 or 332.0) in 2009 but no prior year, and no code for atypical parkinsonism or Lewy body dementia. Controls met these same study eligibility criteria, except that they had no ICD9 code for PD, and were alive in 2009 prior to their randomly assigned reference date (comparable to the cases’ diagnosis dates). The original study included 89,790 cases and 118,095 controls. From this original group of participants, we further restricted to the 48,295 (54%) of cases and 52,324 (44%) of controls who were also enrolled in Medicare Part D and had at least one medication filled under this coverage in 2008–2009. After review of medications taken by the PD patients, we excluded 12,354 cases who had filled a prescription for a medication known to cause secondary parkinsonism (aripiprazole, chlorpromazine, fluphenazine, haloperidol, loxapine, metoclopramide, molindone, olanzapine, paliperidone, perphenazine, pimozide, prochlorperazine, promethazine, quetiapine [if > 100 mg], reserpine, risperidone, tetrabenazine, thioridazine, thiothixene, trifluoperazine, trimethobenzamide and/or ziprasidone) within the 6 months prior to their PD diagnosis in 2009 [15]. This left a total of 35,941 PD cases and 52,324 controls for the present work. We formally divided these participants into a 90% training dataset and 10% test dataset by stratified random sampling (by case status), such that we had 90% cases and 90% controls in our training set for developing the models, and 10% cases and 10% controls in our test set for assessing model performance.

### Calculation of predictor variables

We calculated predictor variables, as previously [13, 16]. In total, during the development of the original predictive model there were 26,468 valid codes (11,063 diagnoses and 15,405 procedures, including ICD9 procedure codes and Healthcare Common Procedure Coding System [HCPCS] codes mainly comprised of Current Procedural Terminology [CPT] codes). CPT codes are part of a formal coding system for billing that encompasses surgical and more minor procedures that physicians perform in the office, along with some radiology and laboratory tests, in contrast to ICD9 procedure codes used by hospitals. HCPCS codes are similar to CPT codes but are specific to Medicare. For ICD9/procedure codes recorded for > 10 PD cases, the median time between receiving the code and PD diagnosis was 2.41 years. This period was nearly identical to the median time for the 536 ICD9/procedure codes selected for our original predictive model ultimately (2.42 years), However, the median time for diagnosis codes indicative of cardinal signs of PD was shorter: 1.51 years for ICD9 333.1 (tremor), 1.98 years for ICD9 781.2 (gait abnormality), 1.09 years for ICD9 781.0 (abnormal involuntary movement), and 1.44 years for ICD9 781.3 (lack of coordination). We calculated age and obtained sex and race/ethnicity from the 2009 beneficiary annual summary file. Given the importance of smoking on PD risk [17], we derived a probability of ever having regularly smoked for each participant using a logistic regression model built from nationwide data [13, 16]. We previously also identified that overall use of medical care is an important predictor of PD and included this variable in our models [13, 18].

Building upon the above data from the beneficiary annual summary file and Part A and B claims that were available to us when we developed our original PD predictive model, we obtained Medicare Part D prescription data from 2008–2009, i.e., in the one to two years prior to PD diagnosis, for use in our predictive models. We derived prescription data from a shorter pre-diagnosis period than for our other claims data because Part D coverage first became available in late 2006. For each medication, we identified all associated active ingredients and created a dichotomous variable representing whether a pharmacy filled a prescription claim for a medication containing the active ingredient at any time during this period prior to the PD diagnosis/control reference date. There were 880 active ingredients represented in these prescription claims data. We did not include 31 active ingredients that could be used to treat PD (carbidopa-levodopa, pramipexole, ropinirole, entacapone, tolcapone, selegiline, rasagline, trihexiphenidyl, benztropine) or that could cause secondary parkinsonism (22 listed above).

### Model building approach

We built all models within the training set (90% stratified random sample) using R version 3.5.0. For all models, we used a two-step model building approach with the same first step for all. In this first step, we identified diagnosis/procedure codes and active ingredients associated with PD using multivariable logistic regression. For each code and active ingredient, we fit a logistic regression model adjusting *a priori* for age (modeled as a two-part linear spline with a knot at age 85), sex, race/ethnicity (7 categories [6 dummy variables]), probability of ever smoking (continuous), and number of unique diagnosis codes (continuous) [18]. These constitute the 11 forced demographic predictors. We then used the Bonferroni correction for multiple comparisons to select a subset of all codes and active ingredients still significantly associated with PD to consider in the second step of the model building. This prescreening retained 983 codes and active ingredients, after we excluded ten that effectively were sex-specific, i.e. acting as a proxy for the patient’s sex.

Starting with the preselected set of predictor variables from the first step, i.e. the 983 codes/active ingredient variables and the 11 forced demographic variables, we proceeded to the second step, which differed for each model. We produced three models (fit three predetermined classifiers): two penalized logistic regression models [13] (one with and one without prescription medications) and a random forest that considered the prescription medications.

For the penalized logistic regressions, we built the models using only the LASSO regression using the R package *glmnet* [19, 20]. In our previous work, we determined that LASSO alone (i.e., α = 1) produced the optimal model as part of the elastic net algorithm [13]. This procedure selects variables and regularizes coefficients based on penalties for possible overfitting. The method is particularly suitable for high dimensional data, using ten-fold cross validation to determine the shrinkage parameter (λ), and improves external validity. We used the area under the receiver operator characteristic curve (AUC) as the measure of model quality for selecting λ.

For the random forest, we used the R packages randomForest [21] and varSelRF [22], which is a variable selection package designed for random forests. Specifically, we used a previously developed variable selection procedure [23]. Briefly, one large random forest was trained on the full 90% training set using all 983 predictors and 11 demographic variables. The predictor importance matrix, which contained the mean, un-scaled decrease in prediction accuracy after variable permutation, was estimated once. Then, the 20% of predictors with the lowest importance were dropped, and a new forest was trained on this smaller subset. The process was repeated iteratively, while always using the original importance matrix, until only two predictor variables remained, i.e., 96 times in the present work. Each smaller subset is contained within all larger subsets, and the predictor subset that generated the lowest “out of bag” error was used to construct the final, predetermined random forest classifier. Random forests have several strengths compared with support vector machines that are beneficial in this application, including: 1) a useful, published feature selection method comparable to the LASSO approach [23]; 2) the ability to handle many categorical and/or irrelevant features; 3) automatic feature relevance determination; and 4) an exceptional generalization performance on a wide range of tasks [24]. The first three of these are critical for our data and goals with this study. Additionally, in other machine learning applications in PD, random forests have consistently performed well [10, 25].

After we completed both the random forest and penalized logistic regression models, we also experimented with using both approaches (penalized regression and random forest) simultaneously to produce a single, combined classifier. For this, we fit a penalized logistic regression model that also used the probability of PD generated by the final random forest as a predictor. The random forest’s probability might be able to act like a case preprocessing filter, allowing the penalized regression to detect more complex relationships akin to the strategy of convolution neural networks [26] and the strategy used in Amoroso et al. (2018) [27]. We again started with the preselected set of predictor variables from the first step but included the prediction probabilities from the final random forest classifier as a variable that could be selected.

Finally, given how close to PD diagnosis the cardinal signs were first coded, we repeated all analyses while utilizing predictor variables that we calculated as of the timepoint one year prior to PD diagnosis/control reference. Specifically, we applied a one-year lag.

### Assessment of model performance

We formally assessed the performance of all models in the test set (10% stratified random sample). We were able to separate the model building step from the model diagnostic step in this way because of the size of the available data, allowing for a clean and straightforward interpretation of the test set, as if it were an external dataset. We applied each of the above models (three primary models and one combined model) to this test dataset. Then, with PD case status in this test set as the gold standard, we used R to calculate three summary measures of model performance [28]: the sensitivity at the cut point that correctly classified the most beneficiaries in the test set, the specificity at that cut point, and the AUC. We also repeated these calculations at Youden’s Index [29], the point at which the sum of sensitivity and specificities is maximized, which is not data dependent. We estimated 95% confidence intervals (CIs) using bootstrapping with 2,000 replicates within the R package *pROC* [30] and validated the results using the Stata command *roctab* [31]. We also calculated the percent of records in the test set classified correctly. As further validation for all models, we calculated Spearman’s rho in the test set between the predicted probabilities of PD for each patient derived from each model. This inter-method reliability approach does not require a true gold standard in order to attempt to validate both methods [32]. We compared the AUCs from the penalized regression with Part D to the one without Part D, to assess whether the inclusion of prescription medication data improved discrimination [33]. Using the same method, we also compared the AUCs from the random forest classifier, as well as the combined model, to the penalized regression with Part D data, to assess whether the application of machine learning improved model performance.

## Results

### Characteristics of cases and controls

We observed all known associations [13] between PD and age, sex, race/ethnicity, and smoking (Table 1). On average, cases were 78.8 years old, and controls were 78.1 years old. Cases had substantially more unique ICD9 codes in the five years prior to PD diagnosis as compared to controls up to their comparable reference date.

**Table 1:** Characteristics of Parkinson disease cases and controls with Medicare Part D coverage, U.S. Medicare 2009, %.

	Cases	Controls
	N = 35,941	N = 52,324
Age, years		
66–69	8.1	16.7
70–74	19.5	28.3
75–79	24.2	22.3
80–84	27.3	19.2
85–90	21.0	13.4
Female	64.7	54.0
Race/ethnicity		
White	86.3	83.7
Black	6.0	7.8
Pacific Islander/other	1.2	1.6
Asian	2.9	3.4
Hispanic	3.1	2.9
Native American	0.3	0.4
Unknown	0.1	0.1
Smoking index ≥ median^a^	41.1	51.5
Age, years, mean (SD)	78.8 (6.1)	78.1 (6.2)
Number of unique ICD9 codes, mean (SD)	99.7 (52.4)	76.3 (46.0)

^a^ Predicted probability of ever smoking divided by the person’s total number of unique diagnosis codes.

Abbreviations: ICD9 = International Classification of Diseases, Ninth Revision, Clinical Modification; SD = standard deviation.

### Characteristics of the models

In the present dataset, the initial penalized logistic regression model, without prescription medications, selected 183 ICD9/procedure codes, in addition to the 11 forced demographic variables for a total of 194 predictors (S1 Table). The second model, which repeated the penalized logistic regression, while including the prescription medications, contained all but two of the ICD9/procedure codes from the first model, as well as 50 additional ICD9/procedure codes and 28 prescription medications for a total of 270 predictors (S1 Table). Insofar as the predictors were the same in both of the penalized regression models, the respective ORs were generally similar.

For the random forest classifier model, the optimal subset of predictors contained 272 predictors: 248 ICD9/procedure codes, 18 active ingredients, and 6 of the 11 basic demographic variables (the two age spline variables, sex, smoking, total count of ICD9 codes, black race) (S1 Table).

**Fig 1:**
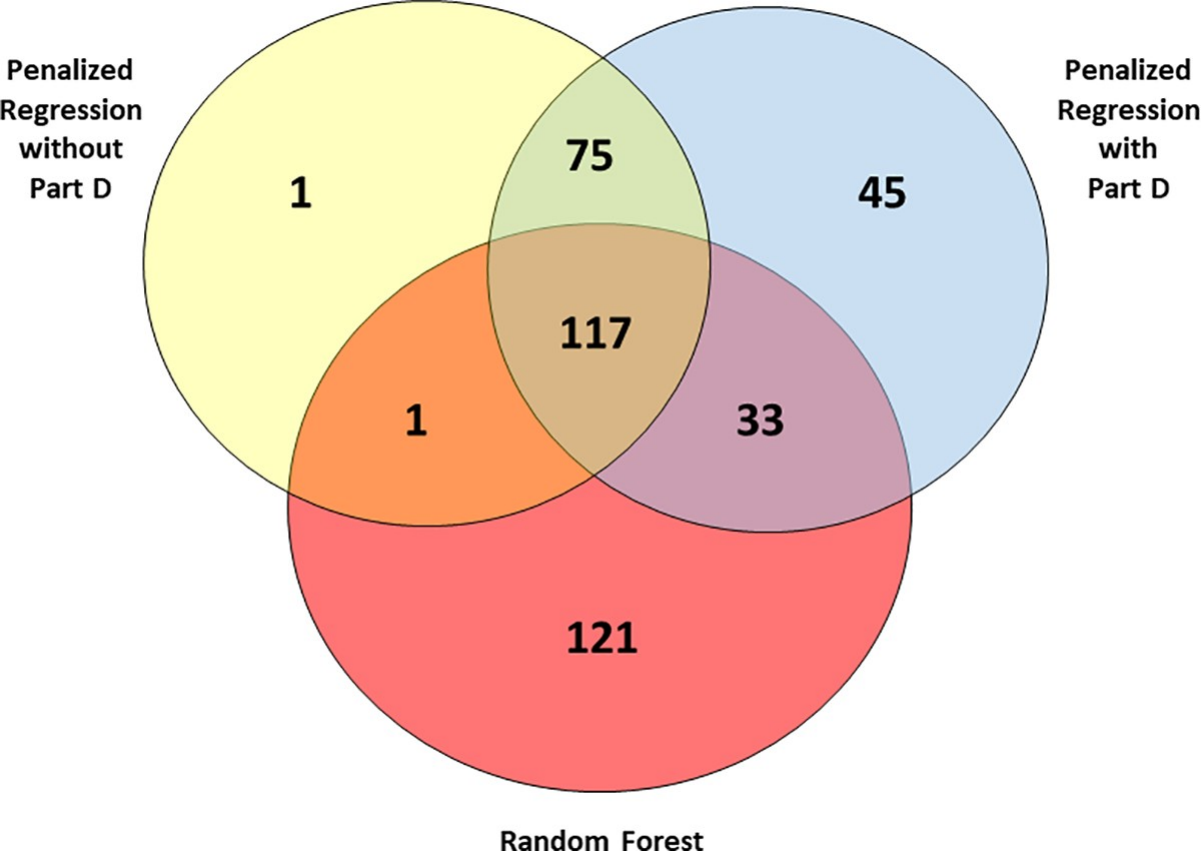
Comparison of distinct and shared predictors between models for predicting Parkinson disease, U.S. Medicare 2009.

Although 121 predictors in the random forest classifier model were not selected into either penalized regression model, there was substantial overlap between the three models in terms of the selected predictors, with 117 predictors (111 ICD9/procedure codes and the above 6 demographic variables) appearing in all three models (Fig 1 and S1 Table). Notably, when we reviewed the non-overlapping codes it was clear that the random forest favored common diagnoses/procedures, including those with modest magnitudes of association with PD, whereas the penalized logistic regression favored rare diagnoses/procedures if the magnitude of the association was relatively large or other uncommon codes. For example, the penalized regression included gout (specifically ICD9 274.9), but the random forest did not.

When we joined the penalized regression and random forest approaches into a combined model, 232 predictors were selected (S2 Table). These predictors included 193 ICD9/procedure codes and 27 prescription medications in addition to the 11 demographic variables and the one variable that captured the predicted probability of PD from the random forest. As expected, we observed the largest OR for the single predictor that represented the random forest PD prediction probability. The combined model included 10 codes not selected by any of the three primary models (S1 and S2 Tables). However, all these codes had ORs close to one.

### Model performance

When we applied each of the three primary models to the test set, the AUC was quite similar for each of the three models (Table 2). Accordingly, the AUC was not significantly improved either by the addition of the Part D data to the penalized regression, or by using random forest methods instead of penalized regression. We achieved a slightly greater AUC with the combined model, in which the penalized regression model with Part D predictors also included the probability of PD for each participant produced by the random forest as a predictor. However, the AUC was not significantly better as compared to the similar model without this predictor.

**Table 2:** Performance of models for predicting Parkinson disease in the test dataset.

	Cut point that maximizes percent accurately classified^a^		Cut point at Youden’s index^a^		Overall performance	Relative performance^b^	
	Sensitivity	Specificity	Sensitivity	Specificity	AUC(95% CI)		
	(95% CI)	(95% CI)	(95% CI)	(95% CI)			
Penalized regression without Part D	65.5 (63.9–67.1)	83.4 (82.4–84.4)	78.0 (76.7–79.3)	73.2 (71.9–74.4)	0.824 (0.815–0.832)	Reference model	--
Penalized regression with Part D	67.2 (65.6–68.7)	82.6 (81.6–83.7)	78.6 (77.2–79.9)	73.3 (72.1–74.6)	0.827 (0.818–0.836)	p = 0.61	Reference model
Random forest (with Part D)	66.3 (64.7–67.8)	82.8 (81.8–83.9)	76.8 (75.4–78.1)	75.0 (73.9–76.2)	0.826 (0.818–0.835)	--	p = 0.90
Combined model (with Part D)^c^	72.9 (71.5–79.6)	79.6 (78.4–80.7)	76.3 (74.9–77.6)	76.3 (75.0–77.4)	0.835 (0.826–0.843)	--	p = 0.23

^a^ Percent sensitivity or specificity, at selected cut points: The cut point that maximizes the percent accurately classified (data dependent) and the cut point at Youden’s index [29] (not data dependent).

^b^ The AUC is a measure of overall model performance, and the presented p-value assesses relative performance of the specified model as compared to the stated reference model using the method of DeLong et al. [33] to obtain the p-value. A p-value < 0.05 indicates that the two AUCs being compared are significantly different. The first comparison tests whether there is a difference in AUC when including Part D prescription medication data in the penalized regression model. The other comparisons test whether there is a difference in the AUCs across the different approaches in which Part D data were included.

^c^ Random forest classifier’s case prediction probability included as a predictor in a new penalized regression model with Part D prescription medication data.

Abbreviations: AUC = area under the receiver operator characteristic curve; CI = confidence interval.

When we applied a one-year lag to the claims data, the lagged penalized logistic regression with Part D data contained 199 ICD9/procedure codes and no medications, while the random forest contained 155 ICD9/procedure codes and five medications. The lagged penalized regression had an AUC of 0.742 (95% CI 0.731–0.753) and the random forest had an AUC of 0.740 (95% CI 0.729–0.751).

The three primary models had similar sensitivity and specificity. At the cut point that maximized the percent of subjects classified correctly, the combined model had greater sensitivity but slightly less specificity than the penalized regression models (Table 2). At the cut point that maximized the sum of sensitivity and specificity (Youden’s index) [29], all models had sensitivity and specificity estimates that were fairly similar (73.2–78.6%), with the combined model maximizing specificity. The number of records correctly classified in the test set was very similar across all models (76.1% for the penalized regression without medications, 76.4% for the penalized regression with medications, 76.0% for the random forest, and 76.9% for the combined model).

### Agreement between predicted probabilities

For each Medicare beneficiary in our dataset, the two penalized regressions’ probabilities were in very close agreement, despite the second model including prescription medication data (Spearman’s rho = 0.995). When we compared the random forest predicted probabilities to those generated by the penalized regression methods, agreement was still high (Spearman’s rho = 0.915 with the model without Part D data and rho = 0.912 with the model with Part D data used as predictors). The combined model had Spearman’s rho’s of 0.96 with all three models.

## Discussion

Identification of people with PD during the prodromal period represents an urgent research priority due to the need to implement neuroprotective therapies earlier in the neurodegenerative process and to prevent disease related morbidity associated with treatable motor symptoms. Our recent, complementary study [14] validated the previous PD predictive model [13], providing evidence that the model is effective and a possible strategy to identify those in the prodromal stage of PD. The current study continues to build upon this work by assessing the value of adding medication data from Medicare Part D to an ICD9/procedure code-based predictive model, as well as applying machine learning methods to further validate and enhance our previous work [13, 14]. The current study suggests prescription medication data would not improve performance of our original predictions had pharmacy data been available for all of the beneficiaries in that sample, because the AUCs between the models with and without pharmacy data were quite similar and not statistically different. However, adding a random forest classifier might slightly improve our model, which had already performed well. Even though the combined model did not have a statistically significantly higher AUC, such a small gain might be difficult to detect even in this large dataset. The latter method, which uses an independent analytic paradigm, also provided confirmation that our previous modeling approach was well suited to developing a predictive algorithm of undiagnosed PD. In addition, the high correlations between model predictions and the consistency of the discriminative ability to detect PD provide evidence that our previous and current models approach the best possible classifier given the Medicare data structure used in this study. Taken together, this further validates our previous predictive model [13].

Interestingly, the addition of medications to the predictive model did not improve the overall model performance consequentially. The addition of medications resulted in a model with 27% more diagnosis/procedure codes. In fact, the addition of prescription medications complicated the model without greatly improving prediction, suggesting that the diagnoses for which the medications were used sufficiently distinguished PD cases from controls. Moreover, generating hypotheses about the point estimate associations with PD for the medications selected by our model may be difficult, since some medications can be used for a variety of medical conditions which may have directionally opposite associations with PD. Nevertheless, most medications identified in the models consistently aligned with potential pharmacological treatment options of medical conditions shared by all models. Our penalized regression model with Medicare Part D confirmed the recently published “protective” association for albuterol (salbutamol) [34]. However, this might reflect the strong inverse association between tobacco smoking and PD [35], given that carvedilol, which has the opposite pharmacologic effect on β2 adreonoreceptors, also was selected as a negative predictor, and both medications are indicated for smoking-related conditions. The random forest did not select these or similar medications related to smoking but alternatively selected chronic ischemic heart disease and a history of myocardial infarction, both strongly associated with smoking. The medications positively associated with PD that remained in the penalized regression model, beyond what was captured via the diagnosis and procedure codes, were primarily those used to treat depression (fluoxetine, duloxetine, mirtazapine, paroxetine, sertraline, and citalopram), reflecting the importance of the non-motor symptoms during the prodromal PD period.

There were some consistent themes to the predictors selected by the different algorithms. Both random forest and penalized regression models highlighted the importance of key predictors of PD, such as age, sex, white vs. black race, smoking, the cardinal motor signs of PD, and dementia/cognitive impairment. The random forest and the respective penalized logistic regression models (with medication data) shared approximately 43% of the predictors, and these models were comprised almost entirely of ICD9/procedure codes. All models identified diagnosis and procedure codes which were suggestive of both motor and non-motor symptoms and medical conditions associated with PD. Motor signs and/or symptoms, such as “abnormal involuntary movement”, “tremor”, “lack of coordination”, and “abnormality of gait” were recognized by all models as important predictors of PD, as expected. Procedure codes shared among all three models included various brain and spine imaging codes, physical therapy, and a variety of non-specific diagnostic tests. These codes likely reflect a combination of diagnostic workup for prodromal PD symptoms and an attempt to treat progressive motor problems with non-pharmacological approaches. The codes indicative of non-motor symptoms that appeared to identify patients with a high probability of PD reflected gastrointestinal dysfunction (constipation), dysautonomia (orthostatic hypotension, dizziness), and cognitive/psychiatric impairments other than general anxiety (memory loss, altered mental status, mental disorder, and depression). Overall, the codes that were common between the three models demonstrate a prodromal disease state characterized by non-motor symptoms, tremor, gait impairment, and an attempt by health care providers to treat or identify the cause of the symptoms.

The random forest tended to select more common predictors with lower magnitude associations. In contrast, the penalized logistic model selected conditions that were uncommon but with a known association with PD, such as gout. Similarly, in our original predictive model using the same regression method but larger sample size, this approach also selected conditions that are rare but have large magnitude associations with PD, such as REM sleep behavior disorder. The random forest model identified a greater number of unique codes than the penalized regression models, yet the conditions/procedures represented by these codes had weaker associations with PD. Many variables with the highest rank in the importance matrix included common medical conditions that may reflect the importance of health care utilization in being diagnosed with PD [18]. Categories distinguishing the random forest model from the penalized regression models included: 1) prescription medications commonly prescribed for bowel and bladder disorders, cognitive impairment/dementia, and psychiatric disorders (e.g., depression and anxiety); 2) codes indicating head and other body trauma, previously identified comorbidities of PD [8]; and 3) codes indicating health care utilization prior to PD diagnosis. These codes provide interesting insight into an alternative approach to predicting PD. The distinct methodologies we used in our study clearly identify marked clinical differences between prodromal PD patients and the general population.

A strength of the study is that there were approximately 133 cases and 194 controls for each predictor considered during the model fitting process. Theoretically, the large sample size to predictor ratio in our models caused our predictions to approach the asymptotically minimum achievable error [36, 37] for classifying PD. For this reason, and because the penalized regression and random forest machine learning are independent analytic approaches, we also combined these into one model by feeding the PD probability from the random forest into the penalized regression model. This approach increased the AUC by approximately 1% in absolute terms. Although this difference may appear small, a 1% improvement might have a meaningful impact on the absolute number of individuals further screened for PD, when applying the predictive algorithm to a large dataset. Additionally, this improvement may be relatively substantial considering the models may already be close to the asymptotic prediction limit. Interestingly, the combined model’s incorporation of the random forest predictions resulted in a discrimination gain by improving its sensitivity, reinforcing the idea that the random forest captured slightly different information about the cases than the penalized regressions. That is, this model gained greater discrimination by improving case identification, and did so only at a small cost to control identification. This is reasonable because the random forest probability acts like a PD case preprocessing filter, improving sensitivity. In practice, all of these models have the advantage of offering users complete flexibility in their application, such that one can balance sensitivity and specificity to customize to each situation.

Despite the many study strengths, there are several potential limitations. First, Medicare is only a population-based health care program for individuals older than 65; therefore, application of this predictive model to younger individuals would not be appropriate. Second, Medicare data are limited to medical claims data, which are filed upon delivery of medical services or filling of prescriptions. Other datasets, such as electronic medical record systems, may have greater data granularity that could be leveraged for even greater model performance. With that said, electronic medical record systems present substantial data quality challenges, as well [38]. Additionally, we only had pharmacy data for the final two years of the five year period prior to PD diagnosis, which may have limited the usefulness of these data. However, these later years are likely to be predictive due to the prodromal period of PD, insofar as patient symptoms lead to new medications being prescribed or patients discontinuing medications due to side effects. Non-pharmacy data in these later years were quite important to our predictive model. Notably, we found that motor signs of PD had large ORs in the penalized regressions and high importance in the random forest. Because these signs and symptoms tend to occur in the later prodromal period, relatively close to PD diagnosis, application of a one-year lag did materially reduce the AUCs for all of our models. These reductions were similar across all models, but discrimination remained quite good. We also note that ICD9 codes in the final three months before PD diagnosis probably were particularly influential in achieving such high AUCs in the unlagged model. There is an increase in the number of diagnoses (ICD9 codes) assigned to patients around the time of PD diagnosis, as patients seek out care for either their symptoms of PD or other medical conditions. The overall number of unique ICD9 codes is an important predictor, in part because of this phenomenon. In addition, we and others have observed a marked spike in traumas, likely due to falls, in the three months prior to PD diagnosis [9], but that increased risk of fractures is evident for six to seven years prior to PD diagnosis. In addition, non-motor symptoms of PD frequently precede the motor symptoms [13]. Thus, we believe that additional lagging would have a diminished influence on AUCs. As such, prediction of PD more than five years prior to diagnosis will be an important goal for future studies. The present work provides a useful foundation for this future work by demonstrating that these predictive models should be attempted in larger datasets, as utilized in our original predictive model of PD, rather than restricted to individuals with pharmacy coverage.

## Supporting information

S1 Table Three primary predictive models, PD predictive model, U.S. Medicare 2009. *HCPCS codes are similar to CPT codes but are specific to Medicare; Abbreviations: CPT = Current Procedural Terminology; HCPCS = Healthcare Common Procedure Coding System*; ICD9 = International Classification of Diseases, Ninth Revision; PD = Parkinson disease.
(PDF)

S2 Table Combined model, PD predictive model, U.S. Medicare 2009. *HCPCS codes are similar to CPT codes but are specific to Medicare; Abbreviations: CPT = Current Procedural Terminology; HCPCS = Healthcare Common Procedure Coding System*; ICD9 = International Classification of Diseases, Ninth Revision; PD = Parkinson disease.
(PDF)

## References

[1] Siderowf A, Jennings D, Eberly S, Oakes D, Hawkins KA, Ascherio A, et al. Impaired olfaction and other prodromal features in the Parkinson At-Risk Syndrome Study. Mov Disord. 2012;27(3):406–412. doi: 10.1002/mds.24892 22237833 PMC6342466

[2] Plouvier AO, Hameleers RJ, van den Heuvel EA, Bor HH, Olde Hartman TC, Bloem BR, et al. Prodromal symptoms and early detection of Parkinson’s disease in general practice: a nested case-control study. Fam Pract. 2014;31(4):373–378. doi: 10.1093/fampra/cmu025 24869632

[3] Goldman JG, Postuma R. Premotor and nonmotor features of Parkinson’s disease. Curr Opin Neurol. 2014;27(4):434–441. doi: 10.1097/WCO.0000000000000112 24978368 PMC4181670

[4] Schrag A, Horsfall L, Walters K, Noyce A, Petersen I. Prediagnostic presentations of Parkinson’s disease in primary care: a case-control study. Lancet Neurol. 2015;14(1):57–64. doi: 10.1016/S1474-4422(14)70287-X 25435387

[5] Gustafsson H, Nordstrom A, Nordstrom P. Depression and subsequent risk of Parkinson disease: A nationwide cohort study. Neurology. 2015;84(24):2422–2429. doi: 10.1212/WNL.0000000000001684 25995056 PMC4478031

[6] Camacho-Soto A, Warden MN, Searles Nielsen S, Salter A, Brody DL, Prather H, et al. Traumatic brain injury in the prodromal period of Parkinson’s disease: A large epidemiological study using medicare data. Ann Neurol. 2017;82(5):744–754. doi: 10.1002/ana.25074 29024046 PMC5812286

[7] Kenborg L, Rugbjerg K, Lee PC, Ravnskjaer L, Christensen J, Ritz B, et al. Head injury and risk for Parkinson disease: results from a Danish case-control study. Neurology. 2015;84(11):1098–1103. doi: 10.1212/WNL.0000000000001362 25681453 PMC4371406

[8] Nystrom H, Nordstrom A, Nordstrom P. Risk of Injurious Fall and Hip Fracture up to 26 y before the Diagnosis of Parkinson Disease: Nested Case-Control Studies in a Nationwide Cohort. PLoS Med. 2016;13(2):e1001954. doi: 10.1371/journal.pmed.1001954 26836965 PMC4737490

[9] Camacho-Soto A, Gross A, Searles Nielsen S, Miller AN, Warden MN, Salter A, et al. Fractures in the prodromal period of Parkinson disease. Neurology. 2020;94(23):e2448–e2456. doi: 10.1212/WNL.0000000000009452 32345729 PMC7455361

[10] Khoury N, Attal F, Amirat Y, Oukhellou L, Mohammed S. Data-Driven Based Approach to Aid Parkinson’s Disease Diagnosis. Sensors (Basel). 2019;19(2). doi: 10.3390/s19020242 30634600 PMC6359125

[11] Cavallo F, Moschetti A, Esposito D, Maremmani C, Rovini E. Upper limb motor pre-clinical assessment in Parkinson’s disease using machine learning. Parkinsonism Relat Disord. 2019;63:111–116. doi: 10.1016/j.parkreldis.2019.02.028 30826265

[12] Pena-Nogales O, Ellmore TM, de Luis-Garcia R, Suescun J, Schiess MC, Giancardo L. Longitudinal Connectomes as a Candidate Progression Marker for Prodromal Parkinson’s Disease. Front Neurosci. 2018;12:967. doi: 10.3389/fnins.2018.00967 30686966 PMC6333847

[13] Searles Nielsen S, Warden MN, Camacho-Soto A, Willis AW, Wright BA, Racette BA. A predictive model to identify Parkinson disease from administrative claims data. Neurology. 2017;89(14):1448–1456. doi: 10.1212/WNL.0000000000004536 28864676 PMC5631173

[14] Faust IM, Racette BA, Searles Nielsen S. Validation of a Parkinson Disease Predictive Model in a Population-Based Study. Parkinsons Dis. 2020;2020:2857608. doi: 10.1155/2020/2857608 32148753 PMC7054801

[15] Lopez-Sendon J, Mena MA, de Yebenes JG. Drug-induced parkinsonism. Expert Opin Drug Saf. 2013;12(4):487–496. doi: 10.1517/14740338.2013.787065 23540800

[16] Centers for Disease Control and Prevention (CDC). BRFSS 2009 Survey Data and Documentation Atlanta: CDC; 2009 [updated December 4, 2014; accessed May 17, 2016, 2016]. Available from: http://www.cdc.gov/brfss/annual_data/annual_2009.htm.

[17] Ritz B, Ascherio A, Checkoway H, Marder KS, Nelson LM, Rocca WA, et al. Pooled analysis of tobacco use and risk of Parkinson disease. Arch Neurol. 2007;64(7):990–997. doi: 10.1001/archneur.64.7.990 17620489

[18] Gross A, Racette BA, Camacho-Soto A, Dube U, Searles Nielsen S. Use of medical care biases associations between Parkinson disease and other medical conditions. Neurology. 2018;90(24):e2155–e2165. doi: 10.1212/WNL.0000000000005678 29743207 PMC5996836

[19] Zou H, Hastie T. Regularization and variable selection via the elastic net. Journal of the Royal Statistical Society Series B-Statistical Methodology. 2005;67:301–320.

[20] Friedman J, Hastie T, Tibshirani R. Regularization Paths for Generalized Linear Models via Coordinate Descent. J Stat Softw. 2010;33(1):1–22. 20808728 PMC2929880

[21] Liaw A, Wiener M. Classification and Regression by RandomForest. R News. 2002;2(3):18–22.

[22] Diaz-Uriarte R. GeneSrF and varSelRF: a web-based tool and R package for gene selection and classification using random forest. BMC Bioinformatics. 2007;8:328. doi: 10.1186/1471-2105-8-328 17767709 PMC2034606

[23] Diaz-Uriarte R, Alvarez de Andres S. Gene selection and classification of microarray data using random forest. BMC Bioinformatics. 2006;7:3. doi: 10.1186/1471-2105-7-3 16398926 PMC1363357

[24] Fernandez-Delgado M, Cernadas E, Barro S, Amorim D. Do we Need Hundreds of Classifiers to Solve Real World Classification Problems? J Machine Learning Research. 2014;15:3133–3181.

[25] Rovini E, Maremmani C, Moschetti A, Esposito D, Cavallo F. Comparative Motor Pre-clinical Assessment in Parkinson’s Disease Using Supervised Machine Learning Approaches. Ann Biomed Eng. 2018;46(12):2057–2068. doi: 10.1007/s10439-018-2104-9 30030773

[26] LeCun Y, Bengio Y, Hinton G. Deep learning. Nature. 2015;521(7553):436–444. doi: 10.1038/nature14539 26017442

[27] Amoroso N, La Rocca M, Monaco A, Bellotti R, Tangaro S. Complex networks reveal early MRI markers of Parkinson’s disease. Med Image Anal. 2018;48:12–24. doi: 10.1016/j.media.2018.05.004 29807313

[28] Steyerberg EW, Vickers AJ, Cook NR, Gerds T, Gonen M, Obuchowski N, et al. Assessing the performance of prediction models: a framework for traditional and novel measures. Epidemiology. 2010;21(1):128–138. doi: 10.1097/EDE.0b013e3181c30fb2 20010215 PMC3575184

[29] Youden WJ. Index for rating diagnostic tests. Cancer. 1950;3(1):32–35. doi: 10.1002/1097-0142(1950)3:1&lt;32::aid-cncr2820030106&gt;3.0.co;2-3 15405679

[30] Robin X, Turck N, Hainard A, Tiberti N, Lisacek F, Sanchez JC, et al. pROC: an open-source package for R and S+ to analyze and compare ROC curves. BMC Bioinformatics. 2011;12:77. doi: 10.1186/1471-2105-12-77 21414208 PMC3068975

[31] StataCorp. Stata MC 14.2. MC 14.2 ed. College Station, TX: StataCorp LP; 2015.

[32] White E, Armstrong BK, Saracci R. Principles of Exposure Measurement in Epidemiology: Collecting, Evaluating, and Improving Measures of Disease Risk Factors. Oxford: Oxford University Press; 2008.

[33] DeLong ER, DeLong DM, Clarke-Pearson DL. Comparing the areas under two or more correlated receiver operating characteristic curves: a nonparametric approach. Biometrics. 1988;44(3):837–845. 3203132

[34] Mittal S, Bjornevik K, Im DS, Flierl A, Dong X, Locascio JJ, et al. beta2-Adrenoreceptor is a regulator of the alpha-synuclein gene driving risk of Parkinson’s disease. Science. 2017;357(6354):891–898. doi: 10.1126/science.aaf3934 28860381 PMC5761666

[35] Searles Nielsen S, Gross A, Camacho-Soto A, Willis AW, Racette BA. beta2-adrenoreceptor medications and risk of Parkinson disease. Ann Neurol. 2018;84(5):683–693. doi: 10.1002/ana.25341 30225948 PMC6881195

[36] Tarca AL, Draghici S, Romero R. Developing classifiers for the detection of cancer using multi-analytes. Methods Mol Biol. 2009;520:259–272. doi: 10.1007/978-1-60327-811-9_19 19381961 PMC3748835

[37] Figueroa RL, Zeng-Treitler Q, Kandula S, Ngo LH. Predicting sample size required for classification performance. BMC Med Inform Decis Mak. 2012;12:8. doi: 10.1186/1472-6947-12-8 22336388 PMC3307431

[38] Harrington L. Copy-Forward in Electronic Health Records: Lipstick on a Pig. Jt Comm J Qual Patient Saf. 2017;43(8):371–374. doi: 10.1016/j.jcjq.2017.04.007 28738981

