# Supplementary material for: A comparison of prediction approaches for identifying prodromal Parkinson disease
Source: PLoS One. 2021 Aug 26;16(8):e0256592. doi: 10.1371/journal.pone.0256592 (PMC8389479; doi:10.1371/journal.pone.0256592)
Supplement: S1 Table — *HCPCS codes are similar to CPT codes but are specific to Medicare; Abbreviations: CPT = Current Procedural Terminology; HCPCS = Healthcare Common Procedure Coding System*; ICD9 = International Classification of Diseases, Ninth Revision; PD = Parkinson disease. (PDF) [file pone.0256592.s001.pdf]

S1 Table: Three Primary Predictive Models, PD Predictive Model, U.S. Medicare 2009

Variable Type	Variable Description	Random Forest Importance	Random Forest Rank	Odds Ratio (Penalized Regression without Part D)	Odds Ratio (Penalized Regression with Part D)	Cases (N)	Controls (N)	Males (N)	Females (N)	Model(s) Including this Predictor	Penalized Regression without Part D	Penalized Regression with Part D	Random Forest	TOTAL PREDICTORS OVERLAPPING ACROSS THESE MODELS
Demographic	Age in years (continuous to age 85)	0.0055	262	1.04	1.04	NA	NA	NA	NA	All Models	1	1	1	
Demographic	Age (number of years after age 85, continuous)	0.0005	166	0.92	0.92	NA	NA	NA	NA	All Models	1	1	1	
Demographic	Count of unique diagnosis codes (continuous)	0.0061	264	1.00	1.00	NA	NA	NA	NA	All Models	1	1	1	
Demographic	Race (black)	0.0001	1	0.73	0.75	2165	4100	2091	4174	All Models	1	1	1	
Demographic	Sex (female)	0.0079	267	0.47	0.47	19409	33827	NA	NA	All Models	1	1	1	
Demographic	Smoking (probability ever/never, continuous)	0.0038	256	0.74	0.75	NA	NA	NA	NA	All Models	1	1	1	
ICD9 diagnosis code	1101 DERMATOPHYTOSIS OF NAIL	0.0014	240	1.16	1.16	14189	12544	9970	16763	All Models	1	1	1	
ICD9 diagnosis code	2859 ANEMIA NOS	0.0007	203	1.01	1.02	18952	20931	14988	24895	All Models	1	1	1	
ICD9 diagnosis code	29410 DEMENTIA W/O BEHAV DIST	0.0027	251	1.02	1.01	5013	2231	2532	4712	All Models	1	1	1	
ICD9 diagnosis code	29411 DEMENTIA W BEHAVIOR DIST	0.0003	100	1.00	0.94	2314	1161	1275	2200	All Models	1	1	1	
ICD9 diagnosis code	2948 MENTAL DISOR NEC OTH DIS	0.0081	268	1.27	1.19	9470	4636	5202	8904	All Models	1	1	1	
ICD9 diagnosis code	2989 PSYCHOSIS NOS	0.0014	242	0.99	0.97	5382	3172	3195	5359	All Models	1	1	1	
ICD9 diagnosis code	311 DEPRESSIVE DISORDER NEC	0.0015	244	1.14	1.07	12561	10434	6866	16129	All Models	1	1	1	
ICD9 diagnosis code	3310 ALZHEIMER'S DISEASE	0.0111	269	1.54	1.40	7810	3522	4099	7233	All Models	1	1	1	
ICD9 diagnosis code	3319 CEREB DEGENERATION NOS	0.0040	258	1.09	1.08	6822	3786	4362	6246	All Models	1	1	1	
ICD9 diagnosis code	3331 TREMOR NEC	0.0225	271	5.30	5.42	6088	1024	2916	4196	All Models	1	1	1	
ICD9 diagnosis code	33390 EXTRAPYRAMIDAL DIS NOS	0.0009	218	1.78	2.11	583	102	279	406	All Models	1	1	1	
ICD9 diagnosis code	33394 RESTLESS LEGS SYNDROME	0.0006	193	1.73	1.79	1496	838	757	1577	All Models	1	1	1	
ICD9 diagnosis code	33399 EXTRAPYRAMIDAL DIS NEC	0.0005	161	1.32	1.35	1720	1167	950	1937	All Models	1	1	1	
ICD9 diagnosis code	34830 ENCEPHALOPATHY NOS	0.0009	219	1.12	1.14	2262	955	1455	1762	All Models	1	1	1	
ICD9 diagnosis code	3569 IDIO PERIPH NEURPTHY NOS	0.0004	155	1.02	1.02	6130	5031	4550	6611	All Models	1	1	1	
ICD9 diagnosis code	3682 DIPLOPIA	0.0002	20	1.03	1.06	1384	997	1061	1320	All Models	1	1	1	
ICD9 diagnosis code	4111 INTERMED CORONARY SYND	0.0002	37	1.02	1.03	4231	4785	4088	4928	All Models	1	1	1	
ICD9 diagnosis code	4139 ANGINA PECTORIS NEC/NOS	0.0003	60	1.03	1.04	7029	8055	6452	8632	All Models	1	1	1	
ICD9 diagnosis code	41401 CRNRY ATHRSCL NATVE VSSL	0.0008	214	1.05	1.06	15395	17359	15748	17006	All Models	1	1	1	
ICD9 diagnosis code	4439 PERIPH VASCULAR DIS NOS	0.0007	201	1.02	1.03	11144	10923	9342	12725	All Models	1	1	1	
ICD9 diagnosis code	4580 ORTHOSTATIC HYPOTENSION	0.0004	152	1.05	1.07	2948	2006	2193	2761	All Models	1	1	1	
ICD9 diagnosis code	56400 CONSTIPATION NOS	0.0012	233	1.16	1.17	11598	10877	8140	14335	All Models	1	1	1	
ICD9 diagnosis code	5990 URIN TRACT INFECTION NOS	0.0004	153	1.07	1.08	20307	23071	12812	30566	All Models	1	1	1	
ICD9 diagnosis code	7030 INGROWING NAIL	0.0006	186	1.03	1.04	6710	6101	4525	8286	All Models	1	1	1	
ICD9 diagnosis code	71516 LOC PRIM OSTEOART-L/LEG	0.0003	97	0.99	0.99	5892	7719	4279	9332	All Models	1	1	1	
ICD9 diagnosis code	7197 DIFFICULTY IN WALKING	0.0014	239	1.08	1.08	9689	6974	5726	10937	All Models	1	1	1	
ICD9 diagnosis code	72402 SPINAL STENOSIS-LUMBAR	0.0006	189	1.05	1.05	6957	6766	5067	8656	All Models	1	1	1	
ICD9 diagnosis code	7242 LUMBAGO	0.0004	130	1.03	1.03	17176	20205	13305	24076	All Models	1	1	1	
ICD9 diagnosis code	72887 MUSCLE WEAKNESS-GENERAL	0.0038	257	1.23	1.23	10940	7247	6598	11589	All Models	1	1	1	
ICD9 diagnosis code	72989 MUSCSKEL SYMPT LIMB NEC	0.0004	126	1.00	1.02	2626	1793	1769	2650	All Models	1	1	1	
ICD9 diagnosis code	73300 OSTEOPOROSIS NOS	0.0005	175	1.03	1.04	12634	16408	4106	24936	All Models	1	1	1	
ICD9 diagnosis code	73390 BONE & CARTILAGE DIS NOS	0.0004	149	0.95	0.95	9358	13795	3917	19236	All Models	1	1	1	
ICD9 diagnosis code	78002 TRANS ALTER AWARENESS	0.0003	116	0.97	0.95	3756	2387	2394	3749	All Models	1	1	1	
ICD9 diagnosis code	7802 SYNCOPE AND COLLAPSE	0.0010	226	1.01	1.01	11350	9622	8135	12837	All Models	1	1	1	
ICD9 diagnosis code	7804 DIZZINESS AND GIDDINESS	0.0010	227	1.08	1.07	16958	17192	12643	21507	All Models	1	1	1	
ICD9 diagnosis code	78079 MALAISE AND FATIGUE NEC	0.0012	235	1.19	1.19	26539	29663	20792	35410	All Models	1	1	1	
ICD9 diagnosis code	78093 MEMORY LOSS	0.0044	260	1.35	1.29	6544	3262	4036	5770	All Models	1	1	1	
ICD9 diagnosis code	78097 ALTERED MENTAL STATUS	0.0041	259	1.19	1.19	6523	3290	3857	5956	All Models	1	1	1	
ICD9 diagnosis code	78099 OTHER GENERAL SYMPTOMS	0.0006	192	0.95	0.94	6192	4670	3950	6912	All Models	1	1	1	
ICD9 diagnosis code	7810 ABN INVOLUN MOVEMENT NEC	0.0355	272	5.47	5.54	8843	1811	4299	6355	All Models	1	1	1	
ICD9 diagnosis code	7812 ABNORMALITY OF GAIT	0.0150	270	1.53	1.53	17219	11752	10653	18318	All Models	1	1	1	
ICD9 diagnosis code	7813 LACK OF COORDINATION	0.0064	266	1.26	1.25	7001	3542	4188	6355	All Models	1	1	1	
ICD9 diagnosis code	78321 ABNORMAL LOSS OF WEIGHT	0.0009	217	1.19	1.18	7727	6566	5222	9071	All Models	1	1	1	
ICD9 diagnosis code	7840 HEADACHE	0.0004	132	0.97	0.96	11812	12403	7767	16448	All Models	1	1	1	
ICD9 diagnosis code	78449 VOICE DISTURBANCE NEC	0.0002	21	1.17	1.20	2074	1842	1687	2229	All Models	1	1	1	
ICD9 diagnosis code	78701 NAUSEA WITH VOMITING	0.0003	67	0.89	0.89	6076	7322	3900	9498	All Models	1	1	1	
ICD9 diagnosis code	78720 DYSPHAGIA NOS	0.0004	124	1.05	1.06	7561	6659	5446	8774	All Models	1	1	1	
ICD9 diagnosis code	78830 URINARY INCONTINENCE NOS	0.0009	221	1.11	1.09	6993	5344	3697	8640	All Models	1	1	1	
ICD9 diagnosis code	78831 URGE INCONTINENCE	0.0003	104	1.13	1.13	3279	2495	1921	3853	All Models	1	1	1	
ICD9 diagnosis code	78841 URINARY FREQUENCY	0.0003	111	1.02	1.02	9216	9086	8481	9821	All Models	1	1	1	
ICD9 diagnosis code	78900 ABDMNAL PAIN UNSPCF SITE	0.0002	57	0.98	0.98	16073	19965	12974	23064	All Models	1	1	1	
ICD9 diagnosis code	7993 DEBILITY NOS	0.0005	176	1.05	1.07	5204	3408	3061	5551	All Models	1	1	1	
ICD9 diagnosis code	E8859 FALL FROM SLIPPING NEC	0.0003	94	1.03	1.03	4030	3210	2023	5217	All Models	1	1	1	**117**
ICD9 diagnosis code	E8889 FALL NOS	0.0006	191	1.06	1.06	6426	4501	3192	7735	All Models	1	1	1	
ICD9 diagnosis code	V1588 PERSONAL HISTORY OF FALL	0.0010	228	1.18	1.18	4195	2079	1957	4317	All Models	1	1	1	
ICD9 diagnosis code	V5481 AFTERCARE JOINT REPLACE	0.0004	143	0.91	0.91	2564	3359	1840	4083	All Models	1	1	1	
HCPCS/CPT procedure code	11721	0.0022	249	1.11	1.12	10330	8099	6798	11631	All Models	1	1	1	
HCPCS/CPT procedure code	20610	0.0004	128	0.99	0.99	9388	12416	7017	14787	All Models	1	1	1	
HCPCS/CPT procedure code	51798	0.0007	198	1.03	1.03	5863	5018	7761	3120	All Models	1	1	1	
HCPCS/CPT procedure code	70450	0.0059	263	1.30	1.29	19157	14857	12900	21114	All Models	1	1	1	
HCPCS/CPT procedure code	70470	0.0005	167	1.16	1.18	2829	2085	1911	3003	All Models	1	1	1	
HCPCS/CPT procedure code	70544	0.0006	185	0.88	0.85	3696	2811	2678	3829	All Models	1	1	1	
HCPCS/CPT procedure code	70551	0.0063	265	1.52	1.52	8012	4379	5067	7324	All Models	1	1	1	
HCPCS/CPT procedure code	70553	0.0029	253	1.16	1.16	8375	5916	5694	8597	All Models	1	1	1	
HCPCS/CPT procedure code	71010	0.0008	213	1.02	1.04	21748	22688	17707	26729	All Models	1	1	1	
HCPCS/CPT procedure code	72100	0.0005	163	1.03	1.04	9846	10423	6846	13423	All Models	1	1	1	
HCPCS/CPT procedure code	72110	0.0003	91	1.09	1.10	6664	7068	4468	9264	All Models	1	1	1	
HCPCS/CPT procedure code	72125	0.0007	202	1.09	1.10	3416	2085	2077	3424	All Models	1	1	1	
HCPCS/CPT procedure code	72131	0.0002	53	1.03	1.04	2219	1736	1512	2443	All Models	1	1	1	
HCPCS/CPT procedure code	72141	0.0003	74	1.01	1.02	3528	3115	2539	4104	All Models	1	1	1	
HCPCS/CPT procedure code	72148	0.0005	173	1.04	1.04	7296	7468	5158	9606	All Models	1	1	1	
HCPCS/CPT procedure code	74022	0.0002	48	0.99	0.98	3833	4231	2921	5143	All Models	1	1	1	
HCPCS/CPT procedure code	76075	0.0005	165	0.99	0.98	8076	12851	2151	18776	All Models	1	1	1	
HCPCS/CPT procedure code	77003	0.0003	106	1.08	1.10	3181	2932	2264	3849	All Models	1	1	1	
HCPCS/CPT procedure code	80061	0.0003	66	0.84	0.83	32155	47242	31532	47865	All Models	1	1	1	
HCPCS/CPT procedure code	82607	0.0029	252	1.20	1.19	17733	15616	12607	20742	All Models	1	1	1	
HCPCS/CPT procedure code	84436	0.0003	102	1.07	1.06	10424	11595	7428	14591	All Models	1	1	1	
HCPCS/CPT procedure code	84443	0.0004	129	1.08	1.09	31383	41482	26501	46364	All Models	1	1	1	
HCPCS/CPT procedure code	84480	0.0002	45	1.04	1.04	4153	4342	2788	5707	All Models	1	1	1	

HCPCS/CPT procedure code	85651	0.0003	108	1.02	1.03	10120	11150	7366	13904	All Models	1	1	1	
HCPCS/CPT procedure code	85652	0.0003	70	1.01	1.02	8351	9248	6117	11482	All Models	1	1	1	
HCPCS/CPT procedure code	86038	0.0003	69	1.02	1.02	6436	7165	4395	9206	All Models	1	1	1	
HCPCS/CPT procedure code	86592	0.0005	184	1.06	1.06	4266	2618	2948	3936	All Models	1	1	1	
HCPCS/CPT procedure code	93306	0.0004	146	1.22	1.23	6266	5931	5267	6930	All Models	1	1	1	
HCPCS/CPT procedure code	93325	0.0003	107	0.98	0.98	20740	24848	18582	27006	All Models	1	1	1	
HCPCS/CPT procedure code	93880	0.0005	168	1.00	0.98	15693	16463	13274	18882	All Models	1	1	1	
HCPCS/CPT procedure code	95861	0.0002	46	1.02	1.02	2930	2274	2150	3054	All Models	1	1	1	
HCPCS/CPT procedure code	95903	0.0003	84	1.01	1.01	4370	3804	3216	4958	All Models	1	1	1	
HCPCS/CPT procedure code	95904	0.0004	142	1.02	1.02	6216	5701	4668	7249	All Models	1	1	1	
HCPCS/CPT procedure code	97001	0.0008	208	1.00	1.01	17072	17685	12487	22270	All Models	1	1	1	
HCPCS/CPT procedure code	97003	0.0003	63	0.97	0.96	5245	3987	3006	6226	All Models	1	1	1	
HCPCS/CPT procedure code	97110	0.0007	207	1.03	1.04	16995	17766	12480	22281	All Models	1	1	1	
HCPCS/CPT procedure code	97112	0.0011	230	1.01	1.01	7974	6005	5123	8856	All Models	1	1	1	
HCPCS/CPT procedure code	97116	0.0015	243	1.04	1.04	6956	4242	3932	7266	All Models	1	1	1	
HCPCS/CPT procedure code	97535	0.0002	26	1.00	0.97	4681	3493	2672	5502	All Models	1	1	1	
HCPCS/CPT procedure code	99222	0.0004	138.5	1.00	0.99	11683	11298	8629	14352	All Models	1	1	1	
HCPCS/CPT procedure code	99223	0.0007	200	1.00	0.99	16115	15961	12729	19347	All Models	1	1	1	
HCPCS/CPT procedure code	99232	0.0005	179	0.99	0.99	20043	20671	16029	24685	All Models	1	1	1	
HCPCS/CPT procedure code	99239	0.0005	170	0.97	0.96	9781	8933	7267	11447	All Models	1	1	1	
HCPCS/CPT procedure code	99244	0.0005	159	1.06	1.06	22668	27673	20269	30072	All Models	1	1	1	
HCPCS/CPT procedure code	99245	0.0003	110	1.05	1.06	12259	12824	10328	14755	All Models	1	1	1	
HCPCS/CPT procedure code	A0425	0.0012	234	1.07	1.07	18631	16928	13222	22337	All Models	1	1	1	
HCPCS/CPT procedure code	A9579	0.0004	138.5	1.37	1.40	2854	2082	2034	2902	All Models	1	1	1	
HCPCS/CPT procedure code	E0143	0.0012	236	1.06	1.05	8089	6569	4655	10003	All Models	1	1	1	
HCPCS/CPT procedure code	E0156	0.0005	183	1.11	1.13	2303	1589	1057	2835	All Models	1	1	1	
HCPCS/CPT procedure code	E0260	0.0003	88	1.00	1.02	2335	1612	1373	2574	All Models	1	1	1	
HCPCS/CPT procedure code	E0627	0.0002	56	1.03	1.13	672	361	368	665	All Models	1	1	1	
HCPCS/CPT procedure code	G0151	0.0012	232	0.93	0.91	11678	9840	6994	14524	All Models	1	1	1	
HCPCS/CPT procedure code	G0154	0.0008	209	0.98	0.98	12880	11942	8700	16122	All Models	1	1	1	
HCPCS/CPT procedure code	J3420	0.0004	131	1.02	1.02	3440	3138	2248	4330	All Models	1	1	1	
HCPCS/CPT procedure code	R0070	0.0008	210	1.00	0.97	3639	2200	1763	4076	All Models	1	1	1	
ICD9 diagnosis code	490 BRONCHITIS NOS	NA	NA	0.997	NA	5671	7482	4828	8325	Lasso without Part D	1	0	0	1
Prescription Medication (Part D)	Acetaminophen	NA	NA	NA	0.96	12104	16320	10438	17986	Lasso with Part D	0	1	0	
Prescription Medication (Part D)	Albuterol	NA	NA	NA	0.95	4750	6799	4294	7255	Lasso with Part D	0	1	0	
Prescription Medication (Part D)	Amlodipine	NA	NA	NA	0.94	6810	10129	6016	10923	Lasso with Part D	0	1	0	
Prescription Medication (Part D)	Amoxicillin	NA	NA	NA	0.96	6713	9881	6604	9990	Lasso with Part D	0	1	0	
Prescription Medication (Part D)	Atorvastatin	NA	NA	NA	0.98	5703	8701	6098	8306	Lasso with Part D	0	1	0	
Prescription Medication (Part D)	Azithromycin	NA	NA	NA	0.93	7483	11323	6734	12072	Lasso with Part D	0	1	0	
Prescription Medication (Part D)	Carvedilol	NA	NA	NA	0.97	2327	3197	2603	2921	Lasso with Part D	0	1	0	
Prescription Medication (Part D)	Dexlansoprazole	NA	NA	NA	0.98	1735	2383	1412	2706	Lasso with Part D	0	1	0	
Prescription Medication (Part D)	Doxycycline	NA	NA	NA	1.00	2385	3432	2438	3379	Lasso with Part D	0	1	0	
Prescription Medication (Part D)	Ezetimibe	NA	NA	NA	0.95	1962	3258	2218	3002	Lasso with Part D	0	1	0	
Prescription Medication (Part D)	Fluoxetine	NA	NA	NA	1.08	1246	1178	771	1653	Lasso with Part D	0	1	0	
Prescription Medication (Part D)	Fluticasone	NA	NA	NA	1.00	2828	4302	2711	4419	Lasso with Part D	0	1	0	
Prescription Medication (Part D)	Hydrochlorothiazide	NA	NA	NA	0.98	7709	13054	6829	13934	Lasso with Part D	0	1	0	
Prescription Medication (Part D)	Oxycodone	NA	NA	NA	0.95	2244	3149	2110	3283	Lasso with Part D	0	1	0	
Prescription Medication (Part D)	Solifenacin	NA	NA	NA	1.00	933	641	502	1072	Lasso with Part D	0	1	0	
Prescription Medication (Part D)	Zoster vaccine (live)	NA	NA	NA	0.92	805	1727	968	1564	Lasso with Part D	0	1	0	
ICD9 diagnosis code	2114 BENIGN NEOPL RECTUM/ANUS	NA	NA	NA	1.00	1497	2442	1827	2112	Lasso with Part D	0	1	0	
ICD9 diagnosis code	2749 GOUT NOS	NA	NA	NA	0.99	3275	4269	4082	3462	Lasso with Part D	0	1	0	
ICD9 diagnosis code	29020 SENILE DELUSION	NA	NA	NA	0.96	812	483	411	884	Lasso with Part D	0	1	0	
ICD9 diagnosis code	44021 ATH EXT NTV AT W CLAUDCT	NA	NA	NA	0.99	3057	3527	3003	3581	Lasso with Part D	0	1	0	
ICD9 diagnosis code	4414 ABDOM AORTIC ANEURYSM	NA	NA	NA	1.00	2173	2538	2795	1916	Lasso with Part D	0	1	0	
ICD9 diagnosis code	47810 OTHER UPPER RESPIRA	NA	NA	NA	0.98	1090	1403	960	1533	Lasso with Part D	0	1	0	
ICD9 diagnosis code	5693 RECTAL & ANAL HEMORRHAGE	NA	NA	NA	1.00	4222	5513	3771	5964	Lasso with Part D	0	1	0	45
ICD9 diagnosis code	5781 BLOOD IN STOOL	NA	NA	NA	1.00	4223	5232	3812	5643	Lasso with Part D	0	1	0	
ICD9 diagnosis code	7019 SKIN HYPERTRO/ATROPH NOS	NA	NA	NA	0.97	1233	2021	1248	2006	Lasso with Part D	0	1	0	
ICD9 diagnosis code	71594 OSTEOARTHROS NOS-HAND	NA	NA	NA	0.98	1571	2273	1019	2825	Lasso with Part D	0	1	0	
ICD9 diagnosis code	8360 TEAR MED MENISC KNEE-CUR	NA	NA	NA	0.99	1180	2045	1101	2124	Lasso with Part D	0	1	0	
ICD9 diagnosis code	V5811 ANTINEOPLASTIC CHEMO ENC	NA	NA	NA	0.99	499	1015	656	858	Lasso with Part D	0	1	0	
HCPCS/CPT procedure code	00810	NA	NA	NA	1.00	4970	6813	4906	6877	Lasso with Part D	0	1	0	
HCPCS/CPT procedure code	11100	NA	NA	NA	1.01	7960	10208	8363	9805	Lasso with Part D	0	1	0	
HCPCS/CPT procedure code	17110	NA	NA	NA	0.99	2564	3877	2650	3791	Lasso with Part D	0	1	0	
HCPCS/CPT procedure code	20600	NA	NA	NA	0.99	1217	1846	894	2169	Lasso with Part D	0	1	0	
HCPCS/CPT procedure code	3111F	NA	NA	NA	1.05	869	379	555	693	Lasso with Part D	0	1	0	
HCPCS/CPT procedure code	36620	NA	NA	NA	0.99	2984	3605	3512	3077	Lasso with Part D	0	1	0	
HCPCS/CPT procedure code	6045F	NA	NA	NA	1.00	438	259	314	383	Lasso with Part D	0	1	0	
HCPCS/CPT procedure code	71260	NA	NA	NA	1.00	6424	7885	5702	8607	Lasso with Part D	0	1	0	
HCPCS/CPT procedure code	76700	NA	NA	NA	0.99	5689	7089	4620	8158	Lasso with Part D	0	1	0	
HCPCS/CPT procedure code	77300	NA	NA	NA	1.00	1035	1647	1419	1263	Lasso with Part D	0	1	0	
HCPCS/CPT procedure code	77427	NA	NA	NA	0.98	911	1505	1284	1132	Lasso with Part D	0	1	0	
HCPCS/CPT procedure code	82570	NA	NA	NA	1.00	7630	10469	8038	10061	Lasso with Part D	0	1	0	
HCPCS/CPT procedure code	86334	NA	NA	NA	1.02	2391	2057	1836	2612	Lasso with Part D	0	1	0	
HCPCS/CPT procedure code	87081	NA	NA	NA	0.99	1265	1682	1066	1881	Lasso with Part D	0	1	0	
HCPCS/CPT procedure code	90818	NA	NA	NA	0.96	1016	640	557	1099	Lasso with Part D	0	1	0	
HCPCS/CPT procedure code	98941	NA	NA	NA	1.00	4220	5899	4136	5983	Lasso with Part D	0	1	0	
HCPCS/CPT procedure code	E0431	NA	NA	NA	0.98	1786	2418	1577	2627	Lasso with Part D	0	1	0	
Demographic	Race (Pacific Islander/other)	NA	NA	0.91	0.92	439	825	580	684	Lasso with Part D / Lasso without Part D	1	1	0	
Demographic	Race (Asian)	NA	NA	1.02	1.02	1029	1799	1197	1631	Lasso with Part D / Lasso without Part D	1	1	0	
Demographic	Race (Hispanic)	NA	NA	0.91	0.90	1112	1530	1018	1624	Lasso with Part D / Lasso without Part D	1	1	0	
Demographic	Race (Native American)	NA	NA	0.90	0.92	123	217	146	194	Lasso with Part D / Lasso without Part D	1	1	0	
Demographic	Race (Unknown)	NA	NA	1.09	1.08	44	58	41	61	Lasso with Part D / Lasso without Part D	1	1	0	
ICD9 diagnosis code	2724 HYPERLIPIDEMIA NEC/NOS	NA	NA	1.00	1.00	26743	38048	26012	38779	Lasso with Part D / Lasso without Part D	1	1	0	
ICD9 diagnosis code	2767 HYPERPOTASSEMIA	NA	NA	0.97	0.96	3592	4156	2992	4756	Lasso with Part D / Lasso without Part D	1	1	0	
ICD9 diagnosis code	27800 OBESITY NOS	NA	NA	0.97	0.97	4054	6341	3510	6885	Lasso with Part D / Lasso without Part D	1	1	0	
ICD9 diagnosis code	38181 DYSFUNCT EUSTACHIAN TUBE	NA	NA	0.96	0.94	1506	2332	1317	2521	Lasso with Part D / Lasso without Part D	1	1	0	
ICD9 diagnosis code	4619 ACUTE SINUSITIS NOS	NA	NA	0.90	0.91	5805	9513	5102	10216	Lasso with Part D / Lasso without Part D	1	1	0	
ICD9 diagnosis code	4659 ACUTE URI NOS	NA	NA	0.93	0.94	9927	14329	8527	15729	Lasso with Part D / Lasso without Part D	1	1	0	
ICD9 diagnosis code	4660 ACUTE BRONCHITIS	NA	NA	0.99	1.00	12039	16673	10579	18133	Lasso with Part D / Lasso without Part D	1	1	0	
ICD9 diagnosis code	4778 ALLERGIC RHINITIS NEC	NA	NA	1.00	0.97	1600	2428	1398	2630	Lasso with Part D / Lasso without Part D	1	1	0	
ICD9 diagnosis code	4779 ALLERGIC RHINITIS NOS	NA	NA	0.99	0.98	7800	11265	6692	12373	Lasso with Part D / Lasso without Part D	1	1	0	

ICD9 diagnosis code	53081 ESOPHAGEAL REFLUX	NA	NA	0.97	0.96	15432	19926	12531	22827	Lasso with Part D / Lasso without Part D	1	1	0
ICD9 diagnosis code	5533 DIAPHRAGMATIC HERNIA	NA	NA	0.96	0.95	6034	7722	4619	9137	Lasso with Part D / Lasso without Part D	1	1	0
ICD9 diagnosis code	56210 DVRTCLO COLON W/O HMRHG	NA	NA	0.93	0.93	11809	16655	11284	17180	Lasso with Part D / Lasso without Part D	1	1	0
ICD9 diagnosis code	56211 DVRTCLI COLON W/O HMRHG	NA	NA	0.99	0.98	2906	3974	2268	4612	Lasso with Part D / Lasso without Part D	1	1	0
ICD9 diagnosis code	59651 HYPERTONICITY OF BLADDER	NA	NA	1.04	1.03	1904	1474	1032	2346	Lasso with Part D / Lasso without Part D	1	1	0
ICD9 diagnosis code	6929 DERMATITIS NOS	NA	NA	0.97	0.97	8186	10547	7347	11386	Lasso with Part D / Lasso without Part D	1	1	0
ICD9 diagnosis code	71947 JOINT PAIN-ANKLE	NA	NA	1.00	0.99	6158	7698	4138	9718	Lasso with Part D / Lasso without Part D	1	1	0
ICD9 diagnosis code	72703 TRIGGER FINGER	NA	NA	0.97	0.94	934	1585	775	1744	Lasso with Part D / Lasso without Part D	1	1	0
ICD9 diagnosis code	72871 PLANTAR FIBROMATOSIS	NA	NA	0.99	0.98	2133	3366	1999	3500	Lasso with Part D / Lasso without Part D	1	1	0
ICD9 diagnosis code	78071 CHRONIC FATIGUE SYNDROME	NA	NA	1.00	1.02	2025	1910	1368	2567	Lasso with Part D / Lasso without Part D	1	1	0
ICD9 diagnosis code	7859 CARDIOVAS SYS SYMP NEC	NA	NA	0.98	0.97	5221	6418	4688	6951	Lasso with Part D / Lasso without Part D	1	1	0
ICD9 diagnosis code	78609 RESPIRATORY ABNORM NEC	NA	NA	0.98	0.98	13372	15655	11578	17449	Lasso with Part D / Lasso without Part D	1	1	0
ICD9 diagnosis code	7862 COUGH	NA	NA	0.95	0.96	16315	20686	13923	23078	Lasso with Part D / Lasso without Part D	1	1	0
ICD9 diagnosis code	78702 NAUSEA ALONE	NA	NA	0.91	0.90	5203	6330	3045	8488	Lasso with Part D / Lasso without Part D	1	1	0
ICD9 diagnosis code	78904 ABDMNAL PAIN LT LWR QUAD	NA	NA	0.98	0.97	2761	3743	2097	4407	Lasso with Part D / Lasso without Part D	1	1	0
ICD9 diagnosis code	V4589 POST-PROC STATES NEC	NA	NA	0.99	0.99	4846	5756	4223	6379	Lasso with Part D / Lasso without Part D	1	1	0
ICD9 diagnosis code	V700 ROUTINE MEDICAL EXAM	NA	NA	0.95	0.95	5989	9804	6574	9219	Lasso with Part D / Lasso without Part D	1	1	0
ICD9 diagnosis code	V726 LABORATORY EXAMINATION	NA	NA	1.00	0.99	3789	5516	2808	6497	Lasso with Part D / Lasso without Part D	1	1	0
ICD9 diagnosis code	V7651 SCREEN MALIG NEOP-COLON	NA	NA	0.99	0.99	6040	10353	6664	9729	Lasso with Part D / Lasso without Part D	1	1	0
HCPCS/CPT procedure code	01810	NA	NA	0.96	0.93	737	1330	766	1301	Lasso with Part D / Lasso without Part D	1	1	0
HCPCS/CPT procedure code	20550	NA	NA	0.98	0.98	2536	3689	1982	4243	Lasso with Part D / Lasso without Part D	1	1	0
HCPCS/CPT procedure code	20605	NA	NA	0.96	0.95	1761	2492	1554	2699	Lasso with Part D / Lasso without Part D	1	1	0
HCPCS/CPT procedure code	3110F	NA	NA	1.07	1.09	889	395	566	718	Lasso with Part D / Lasso without Part D	1	1	0
HCPCS/CPT procedure code	45380	NA	NA	0.98	0.97	4758	7052	4913	6897	Lasso with Part D / Lasso without Part D	1	1	0
HCPCS/CPT procedure code	72193	NA	NA	0.99	0.99	8885	10820	7509	12196	Lasso with Part D / Lasso without Part D	1	1	0
HCPCS/CPT procedure code	73562	NA	NA	0.99	0.99	5945	7691	4182	9454	Lasso with Part D / Lasso without Part D	1	1	0
HCPCS/CPT procedure code	73564	NA	NA	0.97	0.97	4079	5309	2891	6497	Lasso with Part D / Lasso without Part D	1	1	0
HCPCS/CPT procedure code	73630	NA	NA	0.95	0.95	6252	8548	4391	10409	Lasso with Part D / Lasso without Part D	1	1	0
HCPCS/CPT procedure code	73721	NA	NA	0.99	0.98	2851	4167	2117	4901	Lasso with Part D / Lasso without Part D	1	1	0
HCPCS/CPT procedure code	80076	NA	NA	0.94	0.94	14351	19509	13215	20645	Lasso with Part D / Lasso without Part D	1	1	0
HCPCS/CPT procedure code	82150	NA	NA	0.98	0.97	6157	7754	4694	9217	Lasso with Part D / Lasso without Part D	1	1	0
HCPCS/CPT procedure code	83690	NA	NA	0.98	0.99	5836	7200	4375	8661	Lasso with Part D / Lasso without Part D	1	1	0
HCPCS/CPT procedure code	84132	NA	NA	0.95	0.96	6611	8758	5828	9541	Lasso with Part D / Lasso without Part D	1	1	0
HCPCS/CPT procedure code	84460	NA	NA	0.97	0.97	9560	13745	9356	13949	Lasso with Part D / Lasso without Part D	1	1	0
HCPCS/CPT procedure code	85045	NA	NA	1.00	0.98	2372	2766	2034	3104	Lasso with Part D / Lasso without Part D	1	1	0
HCPCS/CPT procedure code	86850	NA	NA	0.98	0.97	2756	3536	2504	3788	Lasso with Part D / Lasso without Part D	1	1	0
HCPCS/CPT procedure code	88305	NA	NA	0.98	0.98	22183	30713	22465	30431	Lasso with Part D / Lasso without Part D	1	1	0
HCPCS/CPT procedure code	88307	NA	NA	0.94	0.94	2555	3996	2090	4461	Lasso with Part D / Lasso without Part D	1	1	0
HCPCS/CPT procedure code	88311	NA	NA	0.97	0.97	3215	3932	2782	4365	Lasso with Part D / Lasso without Part D	1	1	0
HCPCS/CPT procedure code	90732	NA	NA	0.98	0.98	10679	15479	10366	15792	Lasso with Part D / Lasso without Part D	1	1	0
HCPCS/CPT procedure code	90765	NA	NA	0.97	0.96	2690	3222	2179	3733	Lasso with Part D / Lasso without Part D	1	1	0
HCPCS/CPT procedure code	90772	NA	NA	0.99	0.99	8232	10446	7058	11620	Lasso with Part D / Lasso without Part D	1	1	0
HCPCS/CPT procedure code	90774	NA	NA	0.96	0.96	4740	5859	3826	6773	Lasso with Part D / Lasso without Part D	1	1	0
HCPCS/CPT procedure code	90775	NA	NA	0.94	0.94	2256	3164	1902	3518	Lasso with Part D / Lasso without Part D	1	1	0
HCPCS/CPT procedure code	90782	NA	NA	1.00	0.97	2146	2673	1697	3122	Lasso with Part D / Lasso without Part D	1	1	0
HCPCS/CPT procedure code	90784	NA	NA	0.91	0.89	1418	1843	1149	2112	Lasso with Part D / Lasso without Part D	1	1	0
HCPCS/CPT procedure code	93000	NA	NA	1.00	1.01	22038	29640	21258	30420	Lasso with Part D / Lasso without Part D	1	1	0
HCPCS/CPT procedure code	93015	NA	NA	0.99	0.98	9829	13202	10749	12282	Lasso with Part D / Lasso without Part D	1	1	0
HCPCS/CPT procedure code	94060	NA	NA	0.99	1.00	4692	6294	4582	6404	Lasso with Part D / Lasso without Part D	1	1	0
HCPCS/CPT procedure code	94640	NA	NA	0.99	1.00	3382	4664	2994	5052	Lasso with Part D / Lasso without Part D	1	1	0
HCPCS/CPT procedure code	94720	NA	NA	0.99	0.98	3826	5072	3793	5105	Lasso with Part D / Lasso without Part D	1	1	0
HCPCS/CPT procedure code	96413	NA	NA	0.92	0.86	453	1036	569	920	Lasso with Part D / Lasso without Part D	1	1	0
HCPCS/CPT procedure code	99213	NA	NA	0.82	0.80	34396	50197	33491	51102	Lasso with Part D / Lasso without Part D	1	1	0
HCPCS/CPT procedure code	99214	NA	NA	0.98	0.97	32987	47074	31637	48424	Lasso with Part D / Lasso without Part D	1	1	0
HCPCS/CPT procedure code	E1390	NA	NA	0.91	0.91	2819	3659	2529	3949	Lasso with Part D / Lasso without Part D	1	1	0
HCPCS/CPT procedure code	J1100	NA	NA	0.97	0.98	3689	5658	3356	5991	Lasso with Part D / Lasso without Part D	1	1	0
HCPCS/CPT procedure code	J2405	NA	NA	0.95	0.96	4266	6245	3703	6808	Lasso with Part D / Lasso without Part D	1	1	0
HCPCS/CPT procedure code	J3010	NA	NA	0.99	0.99	7109	10189	7361	9937	Lasso with Part D / Lasso without Part D	1	1	0
HCPCS/CPT procedure code	J3301	NA	NA	1.00	0.99	6405	8535	5117	9823	Lasso with Part D / Lasso without Part D	1	1	0
HCPCS/CPT procedure code	J7050	NA	NA	0.99	0.98	2245	2962	2230	2977	Lasso with Part D / Lasso without Part D	1	1	0
HCPCS/CPT procedure code	L3908	NA	NA	0.96	0.93	1281	1871	725	2427	Lasso with Part D / Lasso without Part D	1	1	0
Prescription Medication (Part D)	Duloxetine	0.0002	42	NA	NA	1309	998	655	1652	Random Forest	0	0	1
Prescription Medication (Part D)	Lactulose	0.0002	28	NA	NA	1181	933	824	1290	Random Forest	0	0	1
Prescription Medication (Part D)	Levetiracetam	0.0002	16	NA	NA	599	309	335	573	Random Forest	0	0	1
Prescription Medication (Part D)	Mirtazapine	0.0003	122	NA	NA	1826	1232	973	2085	Random Forest	0	0	1
Prescription Medication (Part D)	Nitrofurantoin	0.0003	78	NA	NA	3125	3156	995	5286	Random Forest	0	0	1
Prescription Medication (Part D)	Oxybutynin	0.0002	24	NA	NA	1633	1398	865	2166	Random Forest	0	0	1
ICD9 diagnosis code	2662 B-COMPLEX DEFIC NEC	0.0003	118	NA	NA	3687	3098	2485	4300	Random Forest	0	0	1
ICD9 diagnosis code	2720 PURE HYPERCHOLESTEROLEM	0.0002	13	NA	NA	21593	30175	20660	31108	Random Forest	0	0	1
ICD9 diagnosis code	2810 PERNICIOUS ANEMIA	0.0003	72	NA	NA	2606	2392	1777	3221	Random Forest	0	0	1
ICD9 diagnosis code	2851 AC POSTHEMORRHAG ANEMIA	0.0002	58	NA	NA	3515	3826	2547	4794	Random Forest	0	0	1
ICD9 diagnosis code	2900 SENILE DEMENTIA UNCOMP	0.0035	255	NA	NA	5681	2804	3007	5478	Random Forest	0	0	1
ICD9 diagnosis code	29010 PRESENILE DEMENTIA	0.0004	137	NA	NA	1413	655	777	1291	Random Forest	0	0	1
ICD9 diagnosis code	29040 VASCULAR DEMENTIA,UNCOMP	0.0008	215	NA	NA	2061	885	1192	1754	Random Forest	0	0	1
ICD9 diagnosis code	2949 MENTAL DISOR NOS OTH DIS	0.0005	178	NA	NA	1703	809	1042	1470	Random Forest	0	0	1
ICD9 diagnosis code	29620 DEPRESS PSYCHOSIS-UNSPEC	0.0002	43	NA	NA	1559	1163	801	1921	Random Forest	0	0	1
ICD9 diagnosis code	29632 RECURR DEPR PSYCHOS-MOD	0.0002	27	NA	NA	1284	992	677	1599	Random Forest	0	0	1
ICD9 diagnosis code	30000 ANXIETY STATE NOS	0.0005	177	NA	NA	8683	8959	4671	12971	Random Forest	0	0	1
ICD9 diagnosis code	30002 GENERALIZED ANXIETY DIS	0.0002	12	NA	NA	2346	2122	1151	3317	Random Forest	0	0	1
ICD9 diagnosis code	3004 DYSTHYMIC DISORDER	0.0003	119	NA	NA	3494	3072	1654	4912	Random Forest	0	0	1
ICD9 diagnosis code	3101 PERSONALITY CHG OTH DIS	0.0002	59	NA	NA	1175	595	725	1045	Random Forest	0	0	1
ICD9 diagnosis code	34290 UNSP HEMIPLGA UNSPF SIDE	0.0003	83	NA	NA	1569	1090	1150	1509	Random Forest	0	0	1
ICD9 diagnosis code	34590 EPILEP NOS W/O INTR EPIL	0.0003	65	NA	NA	1364	836	890	1310	Random Forest	0	0	1
ICD9 diagnosis code	3488 BRAIN CONDITIONS NEC	0.0004	125	NA	NA	2062	1219	1291	1990	Random Forest	0	0	1
ICD9 diagnosis code	3559 MONONEURITIS NOS	0.0003	99	NA	NA	3210	2635	2265	3580	Random Forest	0	0	1
ICD9 diagnosis code	412 OLD MYOCARDIAL INFARCT	0.0003	80	NA	NA	4857	5468	5508	4817	Random Forest	0	0	1
ICD9 diagnosis code	4149 CHR ISCHEMIC HRT DIS NOS	0.0004	134	NA	NA	6708	7067	6816	6959	Random Forest	0	0	1
ICD9 diagnosis code	43411 CRBL EMBLSM W INFRCT	0.0002	7	NA	NA	795	512	585	722	Random Forest	0	0	1
ICD9 diagnosis code	4359 TRANS CEREB ISCHEMIA NOS	0.0007	205	NA	NA	7698	6001	5472	8227	Random Forest	0	0	1
ICD9 diagnosis code	436 CVA	0.0016	245	NA	NA	7798	5583	5547	7834	Random Forest	0	0	1
ICD9 diagnosis code	4370 CEREBRAL ATHEROSCLEROSIS	0.0003	95	NA	NA	2488	1742	1780	2450	Random Forest	0	0	1
ICD9 diagnosis code	4371 AC CEREBROVASC INSUF NOS	0.0013	238	NA	NA	5599	3518	3557	5560	Random Forest	0	0	1
ICD9 diagnosis code	4379 CEREBROVASC DISEASE NOS	0.0003	76	NA	NA	2499	1839	1752	2586	Random Forest	0	0	1

ICD9 diagnosis code	43820 LATE EF-HEMPLGA SIDE NOS	0.0003	64	NA	NA	1866	1260	1367	1759	Random Forest	0	0	1	
ICD9 diagnosis code	4389 LATE EFFECT CV DIS NOS	0.0004	148	NA	NA	2284	1568	1573	2279	Random Forest	0	0	1	
ICD9 diagnosis code	44020 ATHSCL EXTRM NTV ART NOS	0.0004	147	NA	NA	5498	4692	4007	6183	Random Forest	0	0	1	
ICD9 diagnosis code	5119 PLEURAL EFFUSION NOS	0.0004	127	NA	NA	5072	5193	4310	5955	Random Forest	0	0	1	
ICD9 diagnosis code	51889 OTHER LUNG DISEASE NEC	0.0003	92	NA	NA	8066	9334	7215	10185	Random Forest	0	0	1	
ICD9 diagnosis code	5789 GASTROINTEST HEMORR NOS	0.0001	3	NA	NA	4533	4948	3824	5657	Random Forest	0	0	1	
ICD9 diagnosis code	5932 CYST OF KIDNEY, ACQUIRED	0.0002	39	NA	NA	4359	4784	4720	4423	Random Forest	0	0	1	
ICD9 diagnosis code	5939 RENAL & URETERAL DIS NOS	0.0003	82	NA	NA	8034	8552	7637	8949	Random Forest	0	0	1	
ICD9 diagnosis code	59654 NEUROGENIC BLADDER NOS	0.0003	120	NA	NA	1218	739	982	975	Random Forest	0	0	1	
ICD9 diagnosis code	7020 ACTINIC KERATOSIS	0.0003	115	NA	NA	10903	14077	12243	12737	Random Forest	0	0	1	
ICD9 diagnosis code	7038 DISEASES OF NAIL NEC	0.0006	190	NA	NA	4730	4258	3161	5827	Random Forest	0	0	1	
ICD9 diagnosis code	70703 PRESSURE ULCER, LOW BACK	0.0003	61	NA	NA	1212	747	658	1301	Random Forest	0	0	1	
ICD9 diagnosis code	71596 OSTEOARTHROS NOS-L/LEG	0.0003	117	NA	NA	7786	9941	5517	12210	Random Forest	0	0	1	
ICD9 diagnosis code	71945 JOINT PAIN-PELVIS	0.0003	68	NA	NA	12068	12818	7516	17370	Random Forest	0	0	1	
ICD9 diagnosis code	7230 CERVICAL SPINAL STENOSIS	0.0002	40	NA	NA	2064	1813	1660	2217	Random Forest	0	0	1	
ICD9 diagnosis code	72400 SPINAL STENOSIS NOS	0.0003	114	NA	NA	3256	2726	2133	3849	Random Forest	0	0	1	
ICD9 diagnosis code	7282 MUSC DISUSE ATROPHY NEC	0.0002	44	NA	NA	2304	1555	1276	2583	Random Forest	0	0	1	
ICD9 diagnosis code	78009 OTHER ALTER CONSCIOUSNES	0.0013	237	NA	NA	6306	3995	4035	6266	Random Forest	0	0	1	
ICD9 diagnosis code	7801 HALLUCINATIONS	0.0003	93	NA	NA	777	444	406	815	Random Forest	0	0	1	
ICD9 diagnosis code	78039 CONVULSIONS NEC	0.0005	182	NA	NA	3737	2345	2470	3612	Random Forest	0	0	1	
ICD9 diagnosis code	78199 NERVE/MUSCULSKEL SYM NEC	0.0002	32	NA	NA	829	514	530	813	Random Forest	0	0	1	
ICD9 diagnosis code	7830 ANOREXIA	0.0002	35	NA	NA	2439	1850	1362	2927	Random Forest	0	0	1	
ICD9 diagnosis code	7845 SPEECH DISTURBANCE NEC	0.0010	224	NA	NA	2651	1637	1779	2509	Random Forest	0	0	1	
ICD9 diagnosis code	78722 DYSPHAGIA, OROPHARYNGEAL	0.0002	4	NA	NA	1011	453	634	830	Random Forest	0	0	1	
ICD9 diagnosis code	78907 ABDMNAL PAIN GENERALIZED	0.0001	2	NA	NA	5555	6684	4077	8162	Random Forest	0	0	1	
ICD9 diagnosis code	7930 ABN FINDING-SKULL & HEAD	0.0002	8	NA	NA	1307	844	884	1267	Random Forest	0	0	1	
ICD9 diagnosis code	8054 FX LUMBAR VERTEBRA-CLOSE	0.0002	10	NA	NA	1549	1176	753	1972	Random Forest	0	0	1	
ICD9 diagnosis code	8208 FX NECK OF FEMUR NOS-CL	0.0004	136	NA	NA	2451	1780	1061	3170	Random Forest	0	0	1	
ICD9 diagnosis code	920 CONTUSION FACE/SCALP/NCK	0.0005	169	NA	NA	4116	2963	1976	5103	Random Forest	0	0	1	**121**
ICD9 diagnosis code	95901 HEAD INJURY NOS	0.0008	216	NA	NA	5771	3792	3236	6327	Random Forest	0	0	1	
ICD9 diagnosis code	95909 FACE & NECK INJURY	0.0002	55	NA	NA	2676	1889	1520	3045	Random Forest	0	0	1	
ICD9 diagnosis code	95919 TRUNK INJURY-SITES NEC	0.0003	62	NA	NA	2521	1790	1292	3019	Random Forest	0	0	1	
ICD9 diagnosis code	9596 HIP & THIGH INJURY NOS	0.0002	52	NA	NA	2666	1938	1193	3411	Random Forest	0	0	1	
ICD9 diagnosis code	V4365 JOINT REPLACED KNEE	0.0003	73	NA	NA	3102	3913	2272	4743	Random Forest	0	0	1	
ICD9 diagnosis code	V4581 AORTOCORONARY BYPASS	0.0003	71	NA	NA	4251	4694	5626	3319	Random Forest	0	0	1	
ICD9 diagnosis code	V571 PHYSICAL THERAPY NEC	0.0005	158	NA	NA	9501	8992	6123	12370	Random Forest	0	0	1	
ICD9 diagnosis code	V5789 REHABILITATION PROC NEC	0.0005	171	NA	NA	6357	4972	4181	7148	Random Forest	0	0	1	
ICD9 procedure code	8154 TOTAL KNEE REPLACEMENT	0.0002	15	NA	NA	1663	2370	1311	2722	Random Forest	0	0	1	
ICD9 procedure code	8856 CORONAR ARTERIOGR-2 CATH	0.0002	33	NA	NA	3464	4096	3740	3820	Random Forest	0	0	1	
HCPCS/CPT procedure code	11055	0.0002	51	NA	NA	2422	1986	1444	2964	Random Forest	0	0	1	
HCPCS/CPT procedure code	11719	0.0003	109	NA	NA	2495	2030	1509	3016	Random Forest	0	0	1	
HCPCS/CPT procedure code	11720	0.0006	194	NA	NA	4347	3669	2757	5259	Random Forest	0	0	1	
HCPCS/CPT procedure code	17000	0.0004	145	NA	NA	10771	14090	11765	13096	Random Forest	0	0	1	
HCPCS/CPT procedure code	74230	0.0003	89	NA	NA	2416	1496	1784	2128	Random Forest	0	0	1	
HCPCS/CPT procedure code	78478	0.0003	87	NA	NA	13597	17307	13580	17324	Random Forest	0	0	1	
HCPCS/CPT procedure code	78480	0.0004	135	NA	NA	13559	17233	13522	17270	Random Forest	0	0	1	
HCPCS/CPT procedure code	80164	0.0002	31	NA	NA	1106	519	651	974	Random Forest	0	0	1	
HCPCS/CPT procedure code	81001	0.0002	19	NA	NA	21642	26351	17741	30252	Random Forest	0	0	1	
HCPCS/CPT procedure code	82746	0.0019	247	NA	NA	12428	10529	8736	14221	Random Forest	0	0	1	
HCPCS/CPT procedure code	83921	0.0002	6	NA	NA	1593	1050	1138	1505	Random Forest	0	0	1	
HCPCS/CPT procedure code	84165	0.0002	22	NA	NA	5027	4813	3847	5993	Random Forest	0	0	1	
HCPCS/CPT procedure code	84439	0.0003	103	NA	NA	12888	15420	9195	19113	Random Forest	0	0	1	
HCPCS/CPT procedure code	84484	0.0004	150	NA	NA	12210	12923	9491	15642	Random Forest	0	0	1	
HCPCS/CPT procedure code	87086	0.0004	140	NA	NA	16569	18786	10743	24612	Random Forest	0	0	1	
HCPCS/CPT procedure code	90801	0.0010	225	NA	NA	6081	4014	3503	6592	Random Forest	0	0	1	
HCPCS/CPT procedure code	90805	0.0002	29	NA	NA	1156	995	678	1473	Random Forest	0	0	1	
HCPCS/CPT procedure code	92506	0.0002	11	NA	NA	1279	695	804	1170	Random Forest	0	0	1	
HCPCS/CPT procedure code	92526	0.0003	75	NA	NA	1502	833	862	1473	Random Forest	0	0	1	
HCPCS/CPT procedure code	92610	0.0002	25	NA	NA	1665	960	948	1677	Random Forest	0	0	1	
HCPCS/CPT procedure code	93010	0.0003	86	NA	NA	26302	31395	22947	34750	Random Forest	0	0	1	
HCPCS/CPT procedure code	95816	0.0004	141	NA	NA	2823	1385	1711	2497	Random Forest	0	0	1	
HCPCS/CPT procedure code	95819	0.0004	154	NA	NA	3103	1605	1938	2770	Random Forest	0	0	1	
HCPCS/CPT procedure code	95900	0.0002	54	NA	NA	3502	3254	2674	4082	Random Forest	0	0	1	
HCPCS/CPT procedure code	95934	0.0002	23	NA	NA	2203	1749	1590	2362	Random Forest	0	0	1	
HCPCS/CPT procedure code	96118	0.0002	50	NA	NA	1305	549	794	1060	Random Forest	0	0	1	
HCPCS/CPT procedure code	97530	0.0005	162	NA	NA	8693	7138	5487	10344	Random Forest	0	0	1	
HCPCS/CPT procedure code	99233	0.0010	229	NA	NA	13792	13085	10822	16055	Random Forest	0	0	1	
HCPCS/CPT procedure code	99253	0.0005	181	NA	NA	10931	9932	8199	12664	Random Forest	0	0	1	
HCPCS/CPT procedure code	99254	0.0007	195	NA	NA	14078	13330	11012	16396	Random Forest	0	0	1	
HCPCS/CPT procedure code	99285	0.0008	211	NA	NA	20482	21149	16174	25457	Random Forest	0	0	1	
HCPCS/CPT procedure code	99305	0.0003	112	NA	NA	3165	2012	1640	3537	Random Forest	0	0	1	
HCPCS/CPT procedure code	99306	0.0011	231	NA	NA	4960	3017	2605	5372	Random Forest	0	0	1	
HCPCS/CPT procedure code	99309	0.0019	248	NA	NA	5619	3367	2880	6106	Random Forest	0	0	1	
HCPCS/CPT procedure code	99310	0.0005	160	NA	NA	2121	1241	1104	2258	Random Forest	0	0	1	
HCPCS/CPT procedure code	A0427	0.0010	222	NA	NA	13638	12002	9642	15998	Random Forest	0	0	1	
HCPCS/CPT procedure code	A0428	0.0014	241	NA	NA	7815	5231	4522	8524	Random Forest	0	0	1	
HCPCS/CPT procedure code	A0429	0.0007	206	NA	NA	8800	6759	5296	10263	Random Forest	0	0	1	
HCPCS/CPT procedure code	E0163	0.0005	174	NA	NA	4017	3318	2133	5202	Random Forest	0	0	1	
HCPCS/CPT procedure code	E2601	0.0002	47	NA	NA	1034	727	566	1195	Random Forest	0	0	1	
HCPCS/CPT procedure code	G0152	0.0005	172	NA	NA	5478	3884	2858	6504	Random Forest	0	0	1	
HCPCS/CPT procedure code	G0155	0.0005	156	NA	NA	3082	2119	1634	3567	Random Forest	0	0	1	
HCPCS/CPT procedure code	G0156	0.0003	121	NA	NA	4571	3346	2153	5764	Random Forest	0	0	1	
HCPCS/CPT procedure code	G0179	0.0003	85	NA	NA	2860	2055	1589	3326	Random Forest	0	0	1	
HCPCS/CPT procedure code	G0180	0.0007	196	NA	NA	6143	4871	3640	7374	Random Forest	0	0	1	
HCPCS/CPT procedure code	J1030	0.0002	38	NA	NA	4976	6445	3734	7687	Random Forest	0	0	1	
HCPCS/CPT procedure code	K0001	0.0003	105	NA	NA	2064	1605	1093	2576	Random Forest	0	0	1	
HCPCS/CPT procedure code	P9604	0.0007	199	NA	NA	3073	2079	1657	3495	Random Forest	0	0	1	
HCPCS/CPT procedure code	Q0092	0.0008	212	NA	NA	4083	2473	2003	4553	Random Forest	0	0	1	
HCPCS/CPT procedure code	R0075	0.0002	14	NA	NA	1865	1082	873	2074	Random Forest	0	0	1	
HCPCS/CPT procedure code	G0001	0.0003	77	0.997	NA	25761	33137	22672	36226	Random Forest / Lasso without Part D	1	0	1	1
Prescription Medication (Part D)	Citalopram	0.0006	188	NA	1.21	3046	2173	1661	3558	Random Forest / Lasso with Part D	0	1	1	
Prescription Medication (Part D)	Donepezil	0.0055	261	NA	1.32	5977	2700	3266	5411	Random Forest / Lasso with Part D	0	1	1	
Prescription Medication (Part D)	Escitalopram	0.0010	223	NA	1.24	3371	2284	1685	3970	Random Forest / Lasso with Part D	0	1	1	

Prescription Medication (Part D)	Gabapentin	0.0003	123	NA	1.02	3894	3828	2538	5184	Random Forest / Lasso with Part D	0	1	1	
Prescription Medication (Part D)	Meclizine	0.0002	49	NA	1.12	2410	2423	1461	3372	Random Forest / Lasso with Part D	0	1	1	
Prescription Medication (Part D)	Megestrol	0.0005	164	NA	1.25	1445	759	759	1445	Random Forest / Lasso with Part D	0	1	1	
Prescription Medication (Part D)	Memantine	0.0026	250	NA	1.04	3881	1669	2146	3404	Random Forest / Lasso with Part D	0	1	1	
Prescription Medication (Part D)	Paroxetine	0.0002	34	NA	1.13	1645	1471	783	2333	Random Forest / Lasso with Part D	0	1	1	
Prescription Medication (Part D)	Rivastigmine	0.0007	197	NA	1.19	1281	508	671	1118	Random Forest / Lasso with Part D	0	1	1	
Prescription Medication (Part D)	Sertraline	0.0007	204	NA	1.11	3041	2624	1754	3911	Random Forest / Lasso with Part D	0	1	1	
Prescription Medication (Part D)	Tolterodine	0.0003	81	NA	1.12	2145	1644	991	2798	Random Forest / Lasso with Part D	0	1	1	
Prescription Medication (Part D)	Valproic acid	0.0002	36	NA	1.06	1241	527	705	1063	Random Forest / Lasso with Part D	0	1	1	
ICD9 diagnosis code	2639 PROTEIN-CAL MALNUTR NOS	0.0002	41	NA	1.01	2114	1486	1347	2253	Random Forest / Lasso with Part D	0	1	1	
ICD9 diagnosis code	27651 DEHYDRATION	0.0003	98	NA	1.00	7520	6205	4988	8737	Random Forest / Lasso with Part D	0	1	1	
ICD9 diagnosis code	2930 DELIRIUM D/T OTHER COND	0.0004	144	NA	1.00	2455	1412	1536	2331	Random Forest / Lasso with Part D	0	1	1	
ICD9 diagnosis code	33183 MILD COGNITIVE IMPAIREMT	0.0002	5	NA	1.02	815	300	488	627	Random Forest / Lasso with Part D	0	1	1	
ICD9 diagnosis code	43491 CRBL ART OCL NOS W INFRC	0.0017	246	NA	1.00	6434	4259	4547	6146	Random Forest / Lasso with Part D	0	1	1	**33**
ICD9 diagnosis code	43884 ATAXIA	0.0002	18	NA	1.02	947	351	590	708	Random Forest / Lasso with Part D	0	1	1	
ICD9 diagnosis code	95911 INJURY OF CHEST WALL NEC	0.0003	90	NA	1.00	2512	1807	1507	2812	Random Forest / Lasso with Part D	0	1	1	
ICD9 diagnosis code	9599 INJURY-SITE NOS	0.0002	9	NA	1.02	1972	1290	1090	2172	Random Forest / Lasso with Part D	0	1	1	
ICD9 diagnosis code	V1254 HX TIA/STROKE W/O RESID	0.0005	157	NA	1.01	3292	2190	2293	3189	Random Forest / Lasso with Part D	0	1	1	
ICD9 procedure code	3722 LEFT HEART CARDIAC CATH	0.0002	30	NA	1.01	3345	3944	3668	3621	Random Forest / Lasso with Part D	0	1	1	
HCPCS/CPT procedure code	11056	0.0002	17	NA	1.00	2934	2538	1753	3719	Random Forest / Lasso with Part D	0	1	1	
HCPCS/CPT procedure code	17003	0.0003	79	NA	1.01	8343	10457	9636	9164	Random Forest / Lasso with Part D	0	1	1	
HCPCS/CPT procedure code	72170	0.0004	133	NA	0.99	8085	7860	4844	11101	Random Forest / Lasso with Part D	0	1	1	
HCPCS/CPT procedure code	73510	0.0004	151	NA	1.00	9902	9950	5819	14033	Random Forest / Lasso with Part D	0	1	1	
HCPCS/CPT procedure code	77080	0.0005	180	NA	1.00	7276	12873	2505	17644	Random Forest / Lasso with Part D	0	1	1	
HCPCS/CPT procedure code	78465	0.0003	113	NA	1.01	13872	17629	13863	17638	Random Forest / Lasso with Part D	0	1	1	
HCPCS/CPT procedure code	87088	0.0003	101	NA	1.00	9885	10810	5368	15327	Random Forest / Lasso with Part D	0	1	1	
HCPCS/CPT procedure code	90862	0.0003	96	NA	0.97	2852	2118	1636	3334	Random Forest / Lasso with Part D	0	1	1	
HCPCS/CPT procedure code	99307	0.0009	220	NA	0.99	4926	3183	2608	5501	Random Forest / Lasso with Part D	0	1	1	
HCPCS/CPT procedure code	99308	0.0033	254	NA	1.01	7043	4368	3670	7741	Random Forest / Lasso with Part D	0	1	1	
HCPCS/CPT procedure code	P9603	0.0006	187	NA	0.98	3156	2014	1640	3530	Random Forest / Lasso with Part D	0	1	1	
										**TOTAL PREDICTORS IN EACH MODEL**	**194**	**270**	**272**	

Intercept (Penalized Regression without Part D) 0.061834631
Intercept (Penalized Regression with Part D) 0.071994185

Abbreviations:
CPT = Current Procedural Terminology
HCPCS = Healthcare Common Procedure Coding System*
ICD9 = International Classification of Diseases, Ninth Revision
PD = Parkinson Disease

*HCPCS codes are similar to CPT codes but are specific to Medicare
