# Supplementary material for: A comparison of prediction approaches for identifying prodromal Parkinson disease
Source: PLoS One. 2021 Aug 26;16(8):e0256592. doi: 10.1371/journal.pone.0256592 (PMC8389479; doi:10.1371/journal.pone.0256592)
Supplement: S2 Table — *HCPCS codes are similar to CPT codes but are specific to Medicare; Abbreviations: CPT = Current Procedural Terminology; HCPCS = Healthcare Common Procedure Coding System*; ICD9 = International Classification of Diseases, Ninth Revision; PD = Parkinson disease. (PDF) [file pone.0256592.s002.pdf]

S2 Table: Combined Model, PD Predictive Model, U.S. Medicare 2009

Variable Type	Odds Ratio (Combined Model)	Description
Demographic	0.69	Sex (female)
Demographic	0.77	Race (black)
Demographic	0.79	Smoking (probability ever/never, continuous)
Demographic	0.90	Race (Pacific Islander/other)
Demographic	0.90	Race (Hispanic)
Demographic	0.91	Race (Native American)
Demographic	0.94	Age (number of years after age 85, continuous)
Demographic	0.99	Race (unknown)
Demographic	1.00	Total count of unique ICD9 diagnosis codes
Demographic	1.01	Age in years (continuous to age 85)
Demographic	1.03	Race (Asian)
ICD9 diagnosis code	1.64	3331 TREMOR NEC
ICD9 diagnosis code	1.61	33390 EXTRAPYRAMIDAL DIS NOS
ICD9 diagnosis code	1.54	7810 ABN INVOLUN MOVEMENT NEC
ICD9 diagnosis code	1.53	33394 RESTLESS LEGS SYNDROME
ICD9 diagnosis code	1.22	33399 EXTRAPYRAMIDAL DIS NEC
ICD9 diagnosis code	1.14	78449 VOICE DISTURBANCE NEC
ICD9 diagnosis code	1.13	34830 ENCEPHALOPATHY NOS
ICD9 diagnosis code	1.12	V1588 PERSONAL HISTORY OF FALL
ICD9 diagnosis code	1.10	7812 ABNORMALITY OF GAIT
ICD9 diagnosis code	1.10	78321 ABNORMAL LOSS OF WEIGHT
ICD9 diagnosis code	1.10	56400 CONSTIPATION NOS
ICD9 diagnosis code	1.09	72887 MUSCLE WEAKNESS-GENERAL
ICD9 diagnosis code	1.08	78079 MALAISE AND FATIGUE NEC
ICD9 diagnosis code	1.08	78831 URGE INCONTINENCE
ICD9 diagnosis code	1.06	78097 ALTERED MENTAL STATUS
ICD9 diagnosis code	1.06	78830 URINARY INCONTINENCE NOS
ICD9 diagnosis code	1.05	7993 DEBILITY NOS
ICD9 diagnosis code	1.05	1101 DERMATOPHYTOSIS OF NAIL
ICD9 diagnosis code	1.05	73300 OSTEOPOROSIS NOS
ICD9 diagnosis code	1.04	4111 INTERMED CORONARY SYND
ICD9 diagnosis code	1.04	41401 CRNRY ATHRSCL NATVE VSSL
ICD9 diagnosis code	1.04	5990 URIN TRACT INFECTION NOS
ICD9 diagnosis code	1.04	3314 OBSTRUCTIV HYDROCEPHALUS
ICD9 diagnosis code	1.04	4580 ORTHOSTATIC HYPOTENSION
ICD9 diagnosis code	1.04	7813 LACK OF COORDINATION
ICD9 diagnosis code	1.03	59651 HYPERTONICITY OF BLADDER
ICD9 diagnosis code	1.03	E8889 FALL NOS
ICD9 diagnosis code	1.03	78071 CHRONIC FATIGUE SYNDROME
ICD9 diagnosis code	1.03	78093 MEMORY LOSS
ICD9 diagnosis code	1.03	7804 DIZZINESS AND GIDDINESS
ICD9 diagnosis code	1.03	4139 ANGINA PECTORIS NEC/NOS
ICD9 diagnosis code	1.02	72402 SPINAL STENOSIS-LUMBAR
ICD9 diagnosis code	1.02	7197 DIFFICULTY IN WALKING
ICD9 diagnosis code	1.02	78720 DYSPHAGIA NOS
ICD9 diagnosis code	1.02	7030 INGROWING NAIL
ICD9 diagnosis code	1.01	9599 INJURY-SITE NOS
ICD9 diagnosis code	1.01	3682 DIPLOPIA
ICD9 diagnosis code	1.01	70722 PRESSURE ULCER, STAGE II

ICD9 diagnosis code	1.01	4439 PERIPH VASCULAR DIS NOS
ICD9 diagnosis code	1.00	311 DEPRESSIVE DISORDER NEC
ICD9 diagnosis code	1.00	V1254 HX TIA/STROKE W/O RESID
ICD9 diagnosis code	1.00	27651 DEHYDRATION
ICD9 diagnosis code	1.00	95911 INJURY OF CHEST WALL NEC
ICD9 diagnosis code	1.00	3319 CEREB DEGENERATION NOS
ICD9 diagnosis code	1.00	6829 CELLULITIS NOS
ICD9 diagnosis code	1.00	V7651 SCREEN MALIG NEOP-COLON
ICD9 diagnosis code	1.00	4660 ACUTE BRONCHITIS
ICD9 diagnosis code	1.00	V6709 FOLLOW-UP SURGERY NEC
ICD9 diagnosis code	1.00	8360 TEAR MED MENISC KNEE-CUR
ICD9 diagnosis code	0.99	2749 GOUT NOS
ICD9 diagnosis code	0.99	4389 LATE EFFECT CV DIS NOS
ICD9 diagnosis code	0.99	43889 LATE EFFECT CV DIS NEC
ICD9 diagnosis code	0.99	56211 DVRTCLI COLON W/O HMRHG
ICD9 diagnosis code	0.99	72871 PLANTAR FIBROMATOSIS
ICD9 diagnosis code	0.99	2114 BENIGN NEOPL RECTUM/ANUS
ICD9 diagnosis code	0.99	44021 ATH EXT NTV AT W CLAUDCT
ICD9 diagnosis code	0.99	V726 LABORATORY EXAMINATION
ICD9 diagnosis code	0.99	71594 OSTEOARTHROS NOS-HAND
ICD9 diagnosis code	0.99	78900 ABDMNAL PAIN UNSPCF SITE
ICD9 diagnosis code	0.99	7859 CARDIOVAS SYS SYMP NEC
ICD9 diagnosis code	0.99	4781 Other upper respira
ICD9 diagnosis code	0.98	4779 ALLERGIC RHINITIS NOS
ICD9 diagnosis code	0.98	71947 JOINT PAIN-ANKLE
ICD9 diagnosis code	0.98	29411 DEMENTIA W BEHAVIOR DIST
ICD9 diagnosis code	0.98	V4589 POST-PROC STATES NEC
ICD9 diagnosis code	0.98	73390 BONE & CARTILAGE DIS NOS
ICD9 diagnosis code	0.98	7295 PAIN IN LIMB
ICD9 diagnosis code	0.98	V5481 AFTERCARE JOINT REPLACE
ICD9 diagnosis code	0.98	7840 HEADACHE
ICD9 diagnosis code	0.98	78002 TRANS ALTER AWARENESS
ICD9 diagnosis code	0.97	78609 RESPIRATORY ABNORM NEC
ICD9 diagnosis code	0.97	78904 ABDMNAL PAIN LT LWR QUAD
ICD9 diagnosis code	0.97	7019 SKIN HYPERTRO/ATROPH NOS
ICD9 diagnosis code	0.97	4778 ALLERGIC RHINITIS NEC
ICD9 diagnosis code	0.97	53081 ESOPHAGEAL REFLUX
ICD9 diagnosis code	0.97	6929 DERMATITIS NOS
ICD9 diagnosis code	0.96	2989 PSYCHOSIS NOS
ICD9 diagnosis code	0.96	27800 OBESITY NOS
ICD9 diagnosis code	0.96	2767 HYPERPOTASSEMIA
ICD9 diagnosis code	0.95	V700 ROUTINE MEDICAL EXAM
ICD9 diagnosis code	0.95	V5811 ANTINEOPLASTIC CHEMO ENC
ICD9 diagnosis code	0.95	72703 TRIGGER FINGER
ICD9 diagnosis code	0.95	7862 COUGH
ICD9 diagnosis code	0.95	5533 DIAPHRAGMATIC HERNIA
ICD9 diagnosis code	0.95	78099 OTHER GENERAL SYMPTOMS
ICD9 diagnosis code	0.94	56210 DVRTCLO COLON W/O HMRHG
ICD9 diagnosis code	0.94	4659 ACUTE URI NOS
ICD9 diagnosis code	0.94	38181 DYSFUNCT EUSTACHIAN TUBE
ICD9 diagnosis code	0.93	78701 NAUSEA WITH VOMITING
ICD9 diagnosis code	0.92	78702 NAUSEA ALONE
ICD9 diagnosis code	0.92	4619 ACUTE SINUSITIS NOS
ICD9 diagnosis code	1.03	E8859 FALL FROM SLIPPING NEC
HCPCS/CPT procedure code	1.20	A9579

HCPCS/CPT procedure code	1.18	93306
HCPCS/CPT procedure code	1.14	70551
HCPCS/CPT procedure code	1.11	3111F
HCPCS/CPT procedure code	1.09	E0627
HCPCS/CPT procedure code	1.09	E0156
HCPCS/CPT procedure code	1.08	72110
HCPCS/CPT procedure code	1.08	70470
HCPCS/CPT procedure code	1.07	77003
HCPCS/CPT procedure code	1.06	84436
HCPCS/CPT procedure code	1.06	72125
HCPCS/CPT procedure code	1.04	11721
HCPCS/CPT procedure code	1.03	99244
HCPCS/CPT procedure code	1.03	99245
HCPCS/CPT procedure code	1.03	84480
HCPCS/CPT procedure code	1.03	E0143
HCPCS/CPT procedure code	1.03	93000
HCPCS/CPT procedure code	1.02	70450
HCPCS/CPT procedure code	1.02	72100
HCPCS/CPT procedure code	1.02	3110F
HCPCS/CPT procedure code	1.02	78465
HCPCS/CPT procedure code	1.02	85651
HCPCS/CPT procedure code	1.02	86334
HCPCS/CPT procedure code	1.02	J3420
HCPCS/CPT procedure code	1.01	72148
HCPCS/CPT procedure code	1.01	00810
HCPCS/CPT procedure code	1.01	84443
HCPCS/CPT procedure code	1.01	G0156
HCPCS/CPT procedure code	1.01	86038
HCPCS/CPT procedure code	1.01	82607
HCPCS/CPT procedure code	1.00	11100
HCPCS/CPT procedure code	1.00	E0260
HCPCS/CPT procedure code	1.00	72131
HCPCS/CPT procedure code	1.00	00790
HCPCS/CPT procedure code	1.00	94720
HCPCS/CPT procedure code	1.00	73562
HCPCS/CPT procedure code	1.00	74022
HCPCS/CPT procedure code	1.00	94060
HCPCS/CPT procedure code	1.00	99222
HCPCS/CPT procedure code	1.00	77300
HCPCS/CPT procedure code	1.00	11720
HCPCS/CPT procedure code	1.00	90801
HCPCS/CPT procedure code	1.00	98941
HCPCS/CPT procedure code	1.00	97530
HCPCS/CPT procedure code	0.99	Q0092
HCPCS/CPT procedure code	0.99	17110
HCPCS/CPT procedure code	0.99	76700
HCPCS/CPT procedure code	0.99	73721
HCPCS/CPT procedure code	0.99	90772
HCPCS/CPT procedure code	0.99	72193
HCPCS/CPT procedure code	0.99	J1100
HCPCS/CPT procedure code	0.99	J3301
HCPCS/CPT procedure code	0.99	72170
HCPCS/CPT procedure code	0.99	J3010
HCPCS/CPT procedure code	0.99	93325
HCPCS/CPT procedure code	0.99	88305

HCPCS/CPT procedure code	0.99	85045
HCPCS/CPT procedure code	0.98	J7050
HCPCS/CPT procedure code	0.98	36620
HCPCS/CPT procedure code	0.98	86850
HCPCS/CPT procedure code	0.98	97535
HCPCS/CPT procedure code	0.98	90782
HCPCS/CPT procedure code	0.98	82150
HCPCS/CPT procedure code	0.98	87081
HCPCS/CPT procedure code	0.98	93015
HCPCS/CPT procedure code	0.98	99232
HCPCS/CPT procedure code	0.98	45380
HCPCS/CPT procedure code	0.98	73564
HCPCS/CPT procedure code	0.98	84460
HCPCS/CPT procedure code	0.98	99239
HCPCS/CPT procedure code	0.98	88311
HCPCS/CPT procedure code	0.98	20550
HCPCS/CPT procedure code	0.98	90732
HCPCS/CPT procedure code	0.98	E0431
HCPCS/CPT procedure code	0.97	77427
HCPCS/CPT procedure code	0.97	99214
HCPCS/CPT procedure code	0.97	97003
HCPCS/CPT procedure code	0.97	P9603
HCPCS/CPT procedure code	0.97	90774
HCPCS/CPT procedure code	0.96	90765
HCPCS/CPT procedure code	0.96	93880
HCPCS/CPT procedure code	0.96	G0151
HCPCS/CPT procedure code	0.96	J2405
HCPCS/CPT procedure code	0.95	G0154
HCPCS/CPT procedure code	0.95	90775
HCPCS/CPT procedure code	0.95	84132
HCPCS/CPT procedure code	0.95	90862
HCPCS/CPT procedure code	0.95	20605
HCPCS/CPT procedure code	0.94	80076
HCPCS/CPT procedure code	0.94	73630
HCPCS/CPT procedure code	0.94	99307
HCPCS/CPT procedure code	0.93	88307
HCPCS/CPT procedure code	0.93	L3908
HCPCS/CPT procedure code	0.93	01810
HCPCS/CPT procedure code	0.92	99213
HCPCS/CPT procedure code	0.91	80061
HCPCS/CPT procedure code	0.90	E1390
HCPCS/CPT procedure code	0.90	90784
HCPCS/CPT procedure code	0.90	70544
HCPCS/CPT procedure code	0.88	96413
ICD9 procedure code	1.01	3722 LEFT HEART CARDIAC CATH
Prescription Medication (Part D)	1.21	Megestrol
Prescription Medication (Part D)	1.15	Escitalopram
Prescription Medication (Part D)	1.13	Citalopram
Prescription Medication (Part D)	1.10	Meclizine
Prescription Medication (Part D)	1.07	Valproic acid
Prescription Medication (Part D)	1.06	Paroxetine
Prescription Medication (Part D)	1.06	Tolterodine
Prescription Medication (Part D)	1.05	Rivastigmine
Prescription Medication (Part D)	1.04	Sertraline
Prescription Medication (Part D)	1.03	Donepezil

Prescription Medication (Part D)	1.02	Fluoxetine
Prescription Medication (Part D)	1.01	Solifenacin
Prescription Medication (Part D)	1.00	Doxycycline
Prescription Medication (Part D)	1.00	Metronidazole
Prescription Medication (Part D)	0.99	Hydrochlorothiazide
Prescription Medication (Part D)	0.99	Fluticasone
Prescription Medication (Part D)	0.99	Atorvastatin
Prescription Medication (Part D)	0.96	Carvedilol
Prescription Medication (Part D)	0.96	Amlodipine
Prescription Medication (Part D)	0.95	Amoxicillin
Prescription Medication (Part D)	0.95	Ezetimibe
Prescription Medication (Part D)	0.95	Albuterol
Prescription Medication (Part D)	0.95	Acetaminophen
Prescription Medication (Part D)	0.95	Dexlansoprazole
Prescription Medication (Part D)	0.94	Oxycodone
Prescription Medication (Part D)	0.93	Azithromycin
Prescription Medication (Part D)	0.93	Zoster vaccine (live)
Random forest PD case probability	112.36	Predicted probability of PD from the random forest, continuous
Intercept	0.094124445	

Abbreviations:
CPT = Current Procedural Terminology
HCPCS = Healthcare Common Procedure Coding System*
ICD9 = International Classification of Diseases, Ninth Revision
PD = Parkinson disease

*HCPCS codes are similar to CPT codes but are specific to Medicare
